# Unsymmetrical Pd(II) Pincer Complexes with Benzothiazole and Thiocarbamate Flanking Units: Expedient Solvent-Free Synthesis and Anticancer Potential

**DOI:** 10.3390/ijms242417331

**Published:** 2023-12-10

**Authors:** Vladimir A. Kozlov, Diana V. Aleksanyan, Svetlana G. Churusova, Aleksandr A. Spiridonov, Ekaterina Yu. Rybalkina, Evgenii I. Gutsul, Svetlana A. Aksenova, Alexander A. Korlyukov, Alexander S. Peregudov, Zinaida S. Klemenkova

**Affiliations:** 1 A. N. Nesmeyanov Institute of Organoelement Compounds, Russian Academy of Sciences, ul. Vavilova 28, Str. 1, 119334 Moscow, Russia; fos@ineos.ac.ru (V.A.K.); s_churusova@rambler.ru (S.G.C.); alex.aspir@gmail.com (A.A.S.); evgenii@ineos.ac.ru (E.I.G.); aksenova.sa@phystech.edu (S.A.A.); alex@ineos.ac.ru (A.A.K.); asp@ineos.ac.ru (A.S.P.); zklem@ineos.ac.ru (Z.S.K.); 2 Scientific Laboratory “Advanced Composite Materials and Technologies”, Plekhanov Russian University of Economics, Stremyannyi per. 36, 117997 Moscow, Russia; 3 N. N. Blokhin National Medical Research Center of Oncology of the Ministry of Health of the Russian Federation, Kashirskoe shosse 23, 115478 Moscow, Russia; kate_rybalkina@mail.ru

**Keywords:** benzothiazoles, thiocarbamates, pincer complexes, palladium, solid-phase synthesis, cytotoxicity

## Abstract

Driven by the growing threat of cancer, many research efforts are directed at developing new chemotherapeutic agents, where the central role is played by transition metal complexes. The proper ligand design serves as a key factor to unlock the anticancer potential of a particular metal center. Following a recent trend, we have prepared unsymmetrical pincer ligands that combine benzothiazole and thiocarbamate donor groups. These compounds are shown to readily undergo direct cyclopalladation, affording the target *S*,*C*,*N*-type Pd(II) pincer complexes both in solution and in the absence of a solvent. The solid-phase strategy provided the complexes in an efficient and ecologically friendly manner. The resulting palladacycles are fully characterized using nuclear magnetic resonance (NMR) and infrared (IR) spectroscopy and, in one case, by single-crystal X-ray diffraction (XRD). The solvent-free reactions are additionally analyzed by powder XRD. The pincer complexes exhibit remarkable cytotoxicity against several solid and blood cancer cell lines, including human colorectal carcinoma (HCT116), breast cancer (MCF7), prostate adenocarcinoma (PC3), chronic myelogenous leukemia (K562), multiple plasmacytoma (AMO1), and acute lymphoblastic leukemia (H9), with the dimethylamino-substituted derivative being particularly effective. The latter also induced an appreciable level of apoptosis in both parental and doxorubicin-resistant cells K562 and K562/iS9, vindicating the high anticancer potential of this type of palladacycles.

## 1. Introduction

Metal-based chemotherapeutics play a pivotal role in cancer treatment and are still in the spotlight of modern drug discovery and development, being extensively studied at different levels—starting from primary assays to preclinical studies [1,2,3,4,5,6,7,8,9,10,11,12,13]. Continuous efforts in this field are aimed at surmounting the major challenges associated with the application of conventional platinum(II) anticancer agents, such as severe side effects and intrinsic/acquired resistance. A key aspect in the development of new metal-based chemotherapeutics is the choice of an appropriate ligand system that will facilitate unlocking the anticancer potential of a particular metal center. For palladium, the closest platinum congener, increasing popularity in recent years, has been documented for the so-called pincer-type ligands that feature a specific tridentate monoanionic framework [14]. Their main advantage, along with tunability and versatility, is the ability to provide the resulting metal complexes with high thermodynamic stability and controlled kinetic lability. The latter is particularly important for Pd(II) ions, which, despite the obvious similarity in coordination behavior with their Pt(II) counterparts, display much higher rates of ligand-exchange processes [15] and, thus, require stronger kinetic stabilization. It should also be noted that organopalladium species are gaining considerable importance in anticancer drug design [8,16,17,18,19,20,21].

**Figure 1:**
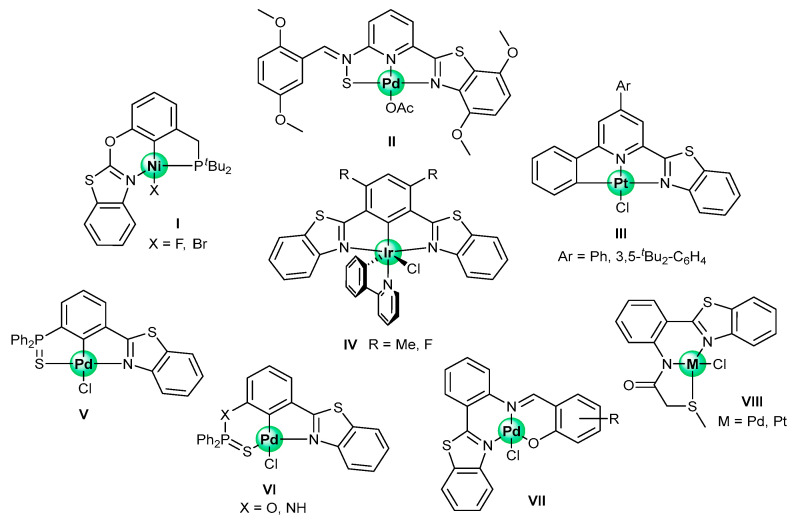
Transition metal pincer complexes with a benzothiazole flanking unit.

Various heterocycles are often used as ligands in the creation of new metal-based therapeutic and diagnostic agents. Owing to a broad spectrum of biological activity (including anticancer, antibacterial, anti-inflammatory, and antiviral properties, not to mention the others), benzothiazole derivatives amount to one of the most extensively studied classes of heterocyclic compounds in medicinal chemistry [22,23,24,25,26,27,28]. Furthermore, a benzothiazole entity is widely represented in different natural products [23]. All this provokes a considerable interest in a benzothiazole scaffold for the design of new metal-based pharmaceuticals. Curiously, transition metal pincer complexes bearing a benzothiazole unit (see, for example, compounds **I**–**VIII** in Figure 1) are largely explored for luminescence properties, electrochemical behavior, and catalytic activity in different reactions [29,30,31,32,33,34,35] but have rarely been tested for their biological effects (for selected reports, see Refs. [36,37,38]). To obtain the related palladium pincer complexes that could be interesting from the viewpoint of further bioactivity studies, we decided to combine a benzothiazole flanking unit with an *S*-donor group, namely, thiocarbamate moiety in a classical benzene-based pincer motif to provide a potentially hemilabile coordination of Pd(II) ions, which can lead to the desired balance between thermodynamic and kinetic stability in the biological medium [14]. The thiocarbamate donor group was chosen to facilitate the solid-phase cyclopalladation of such ligands (vide infra).

In this communication, we report on the synthesis of new pincer ligands combining a benzothiazole unit with a thiocarbamate donor group and the peculiarities of their direct cyclometalation that affords the target Pd(II) complexes. The latter can be obtained not only by the conventional solution-based technique but also according to the recently developed solid-phase methodology in an efficient and ecologically friendly manner. The results of cytotoxicity assays of the cyclopalladated derivatives revealed their high anticancer potential.

## 2. Results and Discussion

**Scheme 1:**
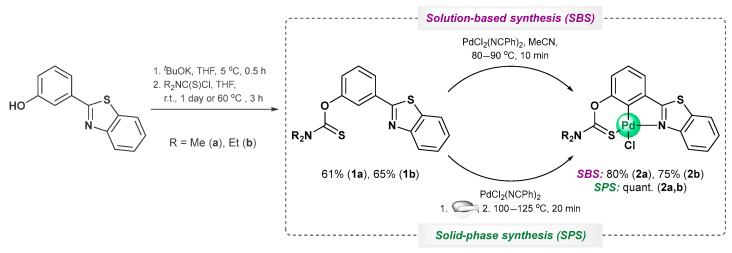
Synthesis of the benzothiazole–thiocarbamate pincer ligands and their direct cyclopalladation in solution and under solvent-free conditions.

A facile synthetic route to the target benzothiazole–thiocarbamate pincer ligands has been devised starting from 3-(benzo[*d*]thiazol-2yl)phenol. The treatment of a potassium salt of this phenol, generated in situ under the action of *^t^*BuOK, with dimethylthiocarbamoyl chloride or its analog having a diethylamino group smoothly afforded compounds **1a**,**b** in good yields (Scheme 1). The key benzothiazole-substituted precursor, in turn, was obtained by the oxidative cyclization of 3-methoxy-*N*-phenylbenzothioamide upon interaction with potassium ferricyanide followed by the demethylation with pyridinium chloride [35].

The resulting ligands readily underwent direct cyclopalladation upon short-term heating with PdCl_2_(NCPh)_2_ in acetonitrile, giving rise to the target pincer-type complexes with five- and six-membered fused metallacycles (Scheme 1). A clear indication of the reaction completion was the light yellow discoloration of the mixture. Note that compounds **2a**,**b** were readily obtained in high yields after simple isolation procedures.

**Figure 2:**
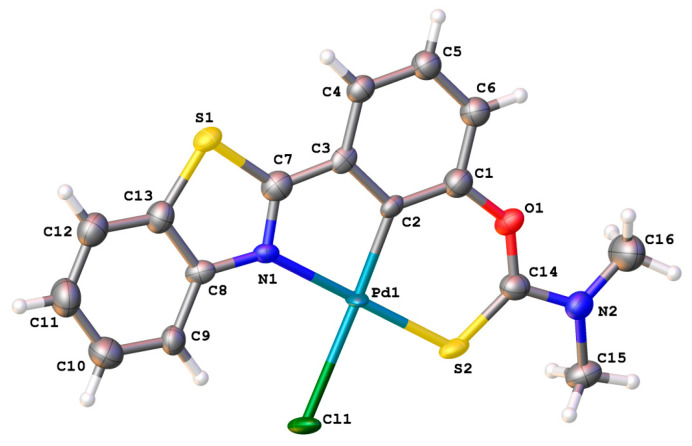
Molecular structure of complex **2a**. The non-hydrogen atoms are drawn as thermal ellipsoids at 40% probability level. The minor component of the disorder and two symmetry-independent molecules of the complex are omitted for clarity.

The realization of a tridentate monoanionic pincer-type coordination in complexes **2a**,**b** was unambiguously confirmed by the NMR and IR spectroscopic data (see Section 3 and Figures S1–S24 in the Supporting Information (SI)). The structure of compound **2a** was corroborated by single-crystal X-ray diffraction analysis (Figure 2). The identities of palladacycles **2a**,**b** were also supported by elemental analyses.

The past decades have been marked by growing attention to different enabling technologies that could mitigate the negative impact of human activity on the environment. This trend rapidly embraced research in chemistry, resulting, in particular, in the development of different sustainable alternatives to conventional solution-based synthetic protocols. A dozen years ago, our research group reported the first example of solid-phase synthesis of Pd(II) pincer complexes based on a series of thiophosphorylated benzothiazole derivatives [39]. This pioneering work laid the foundation for a new, efficient, and ecologically friendly approach to this privileged class of organometallic and metal–organic compounds [40]. Subsequent investigations in the field allowed for extending the solid-phase methodology to other types of pincer systems, including those featuring a central deprotonated secondary amide unit [40,41]. The next major finding appeared to be the first mechanochemical synthesis of pincer complexes realized with a symmetrical bis(thiocarbamate) system [42], which was further adopted for non-classical *N*-metalated species [40] and resonated in the works of other research groups [43,44]. Therefore, it seemed interesting to evaluate the feasibility of the solid-phase synthesis of complexes **2a**,**b**.

The experiments were carried out according to the earlier developed two-step protocol, which included grinding the corresponding ligand and PdCl_2_(NCPh)_2_ in a mortar for several minutes, followed by heating the resulting slightly oily solid residue in an open test tube without the addition of a solvent (Scheme 1). This afforded light yellow free-flowing powders. Note that an optimal heating temperature in each case corresponded to the intensive release of HCl, which was defined with a paper indicator upon heating the mixtures derived after grinding the reactants in a melting point measuring apparatus.

**Figure 3:**
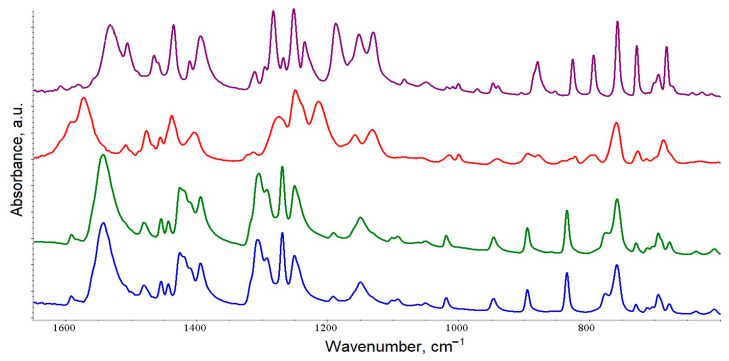
IR spectra of ligand **1a** (violet), a slightly oily solid obtained after grinding **1a** with PdCl_2_(NCPh)_2_ in a mortar (red), a powder obtained after heating of this oily residue (green), and complex **2a** derived from the conventional solution-based synthesis (blue).

The IR spectra of the solid residues obtained after heating (without any workup) replicated those of authentic samples of pincer complexes **2a**,**b** derived from the traditional solution-based synthesis (see Figure 3 and Figures S23–S28 in the SI). The powder X-ray diffraction pattern of compound **2a** derived from the solid-phase synthesis fairly coincided with that of the neat pincer complex obtained in solution (Figure S29 in the SI). At the same time, its counterpart **2b** apparently crystallized in different polymorphic forms under solvent-free and solution-based conditions (Figure S30 in the SI). Nevertheless, indexing and further analysis by the Pawley method (Figures S31 and S32 in the SI) revealed that the calculated XRD patterns fit the experimental ones with quite a high accuracy, indicating the low content of impurities in both samples derived from the solid-phase synthesis. Finally, satisfactory elemental analyses provided by these solids (see Section 3) unambiguously confirmed the high purity of the pincer-type products.

Interestingly, the slightly oily powders, obtained after grinding the reactants in a mortar, unlike their thiophosphorylated analogs [39], did not represent a simple homogenized mixture of the ligand and Pd(II) precursor, consisting mainly of some intermediate non-metalated species and PhCN. Thus, the powder X-ray diffraction analysis revealed that these solid samples are fully amorphous in the case of both benzothiazole–thiocarbamate ligands (see Figures S29 and S30 in the SI). The IR spectra indicated the possible coordination of both thiocarbamate and benzothiazole donor moieties (Figure 3, Figures S27 and S28 in the SI). In addition, the composition of the sample prepared using ligand **1a** and rinsed with hexane appeared to be close to [L·PdCl_2_] (L is the ligand; anal. calcd for L·PdCl_2_: C, 39.08; H, 2.87; N, 5.70; found: C, 40.18; H, 3.32; N, 5.77%). An amorphous nature of these products, i.e., the lack of long-range order, seems to be a key factor that stipulates efficient metalation during heating. Last but not least, the release of benzonitrile in the first step may enable the realization of both the preliminary ligand coordination upon mechanochemical activation and subsequent thermally induced metalation under liquid-assisted conditions. The latter is evidenced by the incomplete cyclopalladation in the sample obtained by grinding ligand **1a** with the Pd(II) precursor and additionally rinsed with hexane upon further heating. Hence, the unsymmetrical palladium(II) pincer complexes featuring benzothiazole and thiocarbamate flanking units can be efficiently produced in the absence of a bulk solvent and readily recovered from the solid-phase synthesis in quantitative yields without any post-synthetic workup.

To estimate the anticancer potential of palladacycles **2a**,**b**, their ability to inhibit cell growth in different solid and blood cancer cell lines was determined using the conventional MTT assay. The results are summarized in Table 1. As can be seen, the diethylamino-substituted derivative (complex **2b**) exhibited remarkable cytotoxic effects on the whole panel of the cancer cell lines explored, being particularly active against plasmacytoma (AMO1) and leukemia (K562 and K562/iS9, H9) cells. In most cases, it was comparable in efficiency to cisplatin, which was used as a positive control in this study. However, on chronic myelogenous leukemia cells (K562 and K562/iS9), palladacycle **2b** significantly outperformed this popular metal-based drug. At the same time, the dimethylamino-substituted analog (complex **2a**) exhibited excellent performance towards all the tested cancer lines. The IC_50_ values for this compound reached up to 0.3 μM in the case of multiple plasmacytoma cells AMO1 and lay in the low micromolar range in the other cases. It is noteworthy that the related thiophosphoryl-substituted benzothiazole derivative featuring two five-membered fused metallacycles (complex **V** in Figure 1 [35]) did not exert any appreciable cell growth inhibitory effects even at a concentration of 80 μM (the percentages of living colon (HCT116), breast (MCF7), and prostate (PC3) cancer cells were over 60%). A benzothiazole derivative bearing a thiophosphoryloxy ancillary donor group (complex **VI** in Figure 1, X = O [35]) demonstrated cytotoxic properties similar to those of palladacycle **2b**. This underscores the importance of a more labile pincer-type coordination that can be achieved not only by the introduction of two different donor centers (like hard nitrogen and soft sulfur atoms) but also via elongating one of the side arms (like in the considered thiophosphoryloxy and thiocarbamate derivatives). In turn, the stability of a pincer-type framework of complex **2b**, used as a representative example, in neat DMSO, DMSO–water, and DMSO–PBS mixtures for 2 days was confirmed by the results of UV-vis spectroscopic studies (for the corresponding spectra, see Figures S33–S35 in the SI). It is noteworthy that free benzothiazole-substituted thiocarbamate **1b** is nontoxic at a concentration as high as 60 μM, which unambiguously indicates that the cytotoxic effects observed for cyclopalladated derivatives **2a**,**b** are mainly due to the coordination by Pd(II) ions.

**Figure 4:**
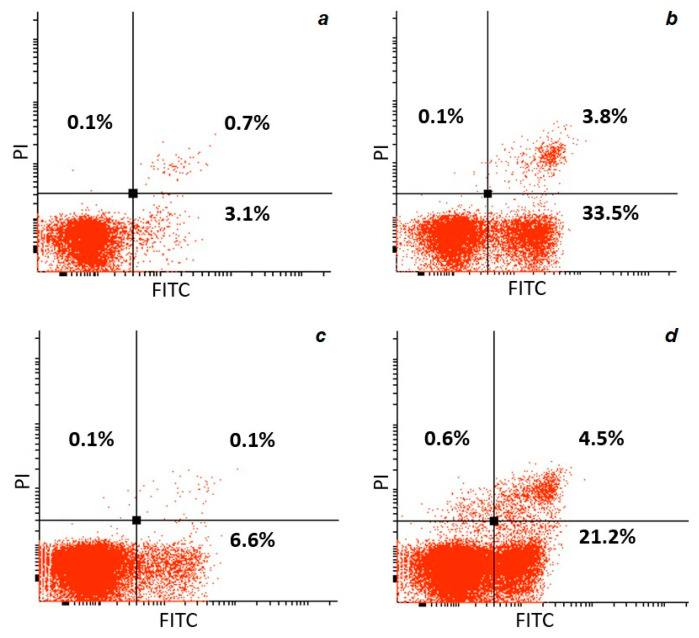
Percentages of necrotic and apoptotic K562 (**a**,**b**) and K562/iS9 (**c**,**d**) cells in the control experiments (**a**,**c**) and after incubation with 1 μM of palladacycle **2a** (**b**,**d**).

It should also be mentioned that, in general, the complexes under consideration exhibited low selectivity to cancer lines, being also toxic towards conditionally normal HEK293 and HBL100 cells. However, in certain cases, the selectivity indices approached and even exceeded 10.0. Of particular note is the same level of cytotoxicity of both benzothiazole–thiocarbamate complexes against parental cell lines and doxorubicin-resistant clones of non-cancerous mammary epithelial cells (HBL100 and HBL100/Dox) and, more importantly, chronic myelogenous leukemia cells K562 and K562/iS9. The additional investigations by flow cytometry performed on K562 and K562/iS9 cancer cells treated with complex **2a** using the Annexin V-FITC/PI double staining assay confirmed its ability to induce perturbation in the cell cycle (Figure 4), increasing the populations of early (lower right quadrants) and late (upper right quadrants) apoptotic cells in both parental and resistant lines. These findings argue for the possibility of creating new promising anticancer agents based on the related cyclopalladated derivatives that could potentially evade drug resistance of cancer cells.

## 3. Materials and Methods

### 3.1. General Remarks

If not mentioned otherwise, all reactions were carried out in the normal atmosphere without taking special precautions to eliminate air and moisture. Tetrahydrofuran was distilled over sodium. Acetonitrile was distilled from P_2_O_5_. 3-(Benzo[*d*]thiazol-2yl)phenol was obtained by the demethylation of 2-(3-methoxyphenyl)benzo[*d*]thiazole under the action of pyridinium chloride according to the earlier described procedure [35]. Diethylthiocarbamoyl chloride was prepared from tetraethylthiuram disulfide upon treatment with sulfuryl chloride [45]. All other chemicals and solvents were used as received.

The NMR spectra were recorded on Bruker Avance 400 and Avance 500 spectrometers (Bruker AXS GmbH, Germany, Karlsruhe), and the chemical shifts (*δ*) were referenced internally by the residual (^1^H) or deuterated (^13^C) solvent signals relative to tetramethylsilane. In most cases, the ^13^C{^1^H} NMR spectra were registered using the *J*MODECHO mode; the signals for the C nuclei bearing odd and even numbers of protons had opposite polarities. The NMR peak assignments for ligand **1a** and complex **2b** were based on the analysis of the ^1^H–^1^H-COSY, ^1^H–^13^C HSQC, and ^1^H–^13^C HMBC spectra. The resulting data were used to assign the NMR spectra of compounds **1b** and **2b**.

The UV–vis spectra were registered on a Cary50 spectrometer in quartz cells with 10 mm path length (Figures S33–S35 in the SI).

The IR spectra were recorded on a Nicolet Magna-IR750 FT spectrometer (resolution 2 cm^−1^, 128 scans). The assignment of absorption bands in the IR spectra was made according to Ref. [46]. Column chromatography was carried out using Macherey-Nagel silica gel 60 (MN Kieselgel 60, 70–230 mesh). Melting points were determined with an MPA 120 EZ-Melt automated melting point apparatus (Stanford Research Systems, Sunnyvale, CA, USA).

### 3.2. Syntheses

#### 3.2.1. *O*-[3-(Benzo[*d*]thiazol-2-yl)phenyl] Dimethylthiocarbamate, **1a**


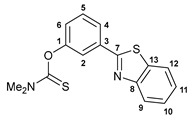


A solution 3-(benzo[*d*]thiazol-2yl)phenol (0.44 g, 1.94 mmol) in THF (10 mL) was added dropwise to a solution of *^t^*BuOK (0.22 g, 1.96 mmol) in THF (5 mL) at 5 °C under an argon atmosphere. The resulting mixture was stirred for 30 min. Then, a solution of dimethylthiocarbamoyl chloride (0.24 g, 1.94 mmol) in THF (10 mL) was added dropwise at 5 °C. The reaction mixture was stirred at 60 °C for 3 h. After cooling to room temperature, the resulting mixture was poured into water. The target product was extracted with EtOAc. The organic layer was separated, dried over anhydrous Na_2_SO_4_, and evaporated to dryness. The residue obtained was treated with hot hexane. The precipitate formed upon cooling was collected by filtration and dried in air to give 0.37 g of the target product as a light yellow crystalline solid. Yield: 61%. Mp: 124–125 °C. ^1^H NMR (500.13 MHz, (CD_3_)_2_SO): *δ* 3.37 and 3.40 (both s, 3H + 3H, NMe_2_), 7.30 (dd, 1H, H(C6), ^3^*J*_HH_ = 8.1 Hz, ^4^*J*_HH_ = 2.2 Hz), 7.48–7.51 (m, 1H, H(C11)), 7.56–7.59 (m, 1H, H(C10)), 7.60–7.63 (m, 1H, H(C5)), 7.81–7.82 (m, 1H, H(C2)), 7.96 (d, 1H, H(C4), ^3^*J*_HH_ = 7.8 Hz), 8.08 (d, 1H, H(C9), ^3^*J*_HH_ = 8.1 Hz), 8.17 (d, 1H, H(C12), ^3^*J*_HH_ = 8.3 Hz) ppm. ^13^C{^1^H} NMR (125.76 MHz, (CD_3_)_2_SO): *δ* 39.13 and 43.42 (both s, NMe_2_), 121.73 (s, C2), 122.95 (s, C12), 123.48 (s, C9), 125.10 (s, C4), 126.25 and 126.35 (both s, C6 and C11), 127.29 (s, C10), 130.77 (s, C5), 134.31 (s, C3), 135.02 (s, C13), 153.90 (s, C8), 154.81 (s, C1), 166.77 (s, C7), 186.52 (s, C=S) ppm. IR (KBr, *ν*/cm^−1^): 682(m), 695(w), 728(m), 757(s), 794(m), 826(m), 880(m), 948(w), 1001(w), 1050(w), 1083(w), 1131(s), 1153(s), 1188(s), 1236(m), 1252(s), 1268(m), 1284(s), 1312(w), 1395(s), 1411(m), 1436(s), 1466(m), 1507(m), 1533(br, s), 1582(w), 2873(vw), 2938(w), 3063(w). Anal. Cacld for C_16_H_14_N_2_OS_2_: C, 61.12; H, 4.49; N, 8.91. Found: C, 61.24; H, 4.58; N, 8.97%.

#### 3.2.2. *O*-[3-(Benzo[*d*]thiazol-2-yl)phenyl] Diethylthiocarbamate, **1b**


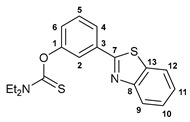


A solution 3-(benzo[*d*]thiazol-2yl)phenol (0.58 g, 2.55 mmol) in THF (15 mL) was added dropwise to a solution of *^t^*BuOK (0.29 g, 2.58 mmol) in THF (15 mL) at 5 °C under an argon atmosphere. The resulting mixture was stirred for 30 min. Then, a solution of diethylthiocarbamoyl chloride (0.39 g, 2.57 mmol) in THF (15 mL) was added dropwise at 5 °C. The reaction mixture was stirred at room temperature for 1 h and left overnight. Then, it was poured into water, and the target product was extracted with Et_2_O. The organic layer was separated, dried over anhydrous Na_2_SO_4_, and evaporated to dryness. The residue obtained was purified by column chromatography (eluent: CH_2_Cl_2_–hexane (1:2)) to give 0.57 g of the target product as light yellow crystals. Yield: 65%. Mp: 135–137 °C. ^1^H NMR (400.13 MHz, CDCl_3_): *δ* 1.35–1.40 (m, 6H, Me), 3.75 (q, 2H, CH_2_, ^3^*J*_HH_ = 7.0 Hz), 3.94 (q, 2H, CH_2_, ^3^*J*_HH_ = 7.0 Hz), 7.25 (dd, 1H, H(C6), ^3^*J*_HH_ = 8.0 Hz, ^4^*J*_HH_ = 1.4 Hz), 7.40–7.44 (m, 1H, H_Ar_), 7.50–7.56 (m, 2H, H_Ar_), 7.88–7.89 (m, 1H, H(C2)), 7.93 (d, 1H, H_Ar_, ^3^*J*_HH_ = 8.0 Hz), 7.96 (d, 1H, H_Ar_, ^3^*J*_HH_ = 7.9 Hz), 8.09 (d, 1H, H_Ar_, ^3^*J*_HH_ = 7.9 Hz) ppm. ^13^C{^1^H} NMR (100.61 MHz, CDCl_3_): *δ* 11.84 and 13.63 (both s, Me), 44.39 and 48.49 (both s, CH_2_), 121.71 and 121.74 (both s, C2 and C12), 123.34 (s, C9), 125.10, 125.41, and 125.61 (three s, C4, C6, and C11), 126.45 (s, C10), 129.68 (s, C5), 134.80 and 135.11 (both s, C3 and C13), 154.00 and 154.40 (both s, C1 and C8), 166.98 (s, C7), 186.48 (s, C=S) ppm. IR (KBr, *ν*/cm^−1^): 680(m), 703(w), 730(m), 756(m), 789(w), 814(w), 887(w), 911(w), 946(w), 1001(w), 1076(w), 1088(w), 1097(w), 1125(m), 1151(m), 1161(m), 1177(s), 1231(s), 1258(m), 1269(m), 1289(m), 1316(m), 1349(w), 1362(w), 1380(w), 1427(m), 1436(s), 1466(m), 1508(s), 1583(w), 2874(vw), 2936(w), 2977(w), 3054(vw). Anal. Cacld for C_18_H_18_N_2_OS_2_: C, 63.13; H, 5.30; N, 8.18. Found: C, 63.19; H, 5.21; N, 8.14%.

#### 3.2.3. Solution-Based Synthesis and Characterization of [*κ*^3^-*S*,*C*,*N*-(L)Pd(II)Cl] Complex **2a**


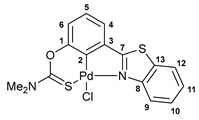


A solution of ligand **1a** (46 mg, 0.146 mmol) and PdCl_2_(NCPh)_2_ (56 mg, 0.146 mmol) in acetonitrile (mL) was heated at 80 °C (oil bath) for 10 min until light yellow discoloration and the formation of a precipitate. The latter was filtered off, washed with Et_2_O, and dried in air to give 53 mg of the target complex as a light yellow crystalline solid. Yield: 80%. ^1^H NMR (500.13 MHz, (CD_3_)_2_SO): *δ* 3.43 and 3.45 (both s, 3H + 3H, NMe_2_), 7.23 (d, 1H, H(C6), ^3^*J*_HH_ = 8.1 Hz), 7.30–7.33 (m, 1H, H(C5)), 7.52–7.55 (m, 1H, H(C11)), 7.57–7.60 (m, 1H, H(C10)), 7.72 (d, 1H, H(C4), ^3^*J*_HH_ = 7.3 Hz), 8.21 (d, 1H, H(C12), ^3^*J*_HH_ = 8.0 Hz), 9.41 (d, 1H, H(C9), ^3^*J*_HH_ = 8.3 Hz) ppm. ^13^C{^1^H} NMR (125.76 MHz, (CD_3_)_2_SO): *δ* 40.69 and 43.43 (both s, NMe_2_), 121.07 (s, C6), 123.35 (s, C9), 123.49 (s, C12), 124.69 (s, C4), 126.62 (s, C11), 127.42 (s, C5), 127.71 (s, C10), 131.43 (s, C3), 131.78 (s, C13), 142.98 (s, C2), 151.09 (s, C8), 151.27 (s, C1), 172.36 (s, C=S), 177.34 (s, C7) ppm. IR (KBr, *ν*/cm^−1^): 678(w), 695(w), 729(w), 758(m), 778(w), 835(m), 895(w), 947(w), 1019(w), 1093(w), 1150 (br, m), 1192(w), 1251(m), 1270(s), 1293(m), 1308(s), 1395(m), 1427(s), 1444(m), 1455(m), 1481(m), 1543 (br, s), 1592(w), 2862(vw), 2937(vw), 3056(w). Anal. Cacld for C_16_H_13_ClN_2_OPdS_2_: C, 42.21; H, 2.88; N, 6.15. Found: C, 42.07; H, 2.94; N, 6.22%.

#### 3.2.4. Solution-Based Synthesis and Characterization of [*κ*^3^-*S*,*C*,*N*-(L)Pd(II)Cl] Complex **2b**


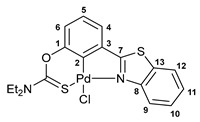


A solution of ligand **1b** (56 mg, 0.164 mmol) and PdCl_2_(NCPh)_2_ (63 mg, 0.164 mmol) in acetonitrile (10 mL) was heated at 90 °C (oil bath) for 10 min. After cooling to room temperature, the resulting light yellow solution was half evaporated until a precipitate formed. Then, Et_2_O (5 mL) was added to the mixture for complete precipitation of the target product. The resulting precipitate was filtered off, washed with Et_2_O, and dried in air to give 59 mg of palladacycle **2b** as a light yellow crystalline solid. Yield: 75%. ^1^H NMR (400.13 MHz, CDCl_3_): *δ* 1.37 (t, 3H, Me, ^3^*J*_HH_ = 7.2 Hz), 1.41 (t, 3H, Me, ^3^*J*_HH_ = 7.2 Hz), 3.75 (q, 2H, CH_2_, ^3^*J*_HH_ = 7.2 Hz), 3.94 (q, 2H, CH_2_, ^3^*J*_HH_ = 7.2 Hz), 6.89 (d, 1H, H(C6), ^3^*J*_HH_ = 8.1 Hz), 7.06–7.10 (m, 1H, H(C5)), 7.38–7.43 (m, 2H, H_Ar_), 7.50–7.54 (m, 1H, H_Ar_), 7.80 (d, 1H, H(C12), ^3^*J*_HH_ = 8.0 Hz), 9.52 (d, 1H, H(C9), ^3^*J*_HH_ = 8.3 Hz) ppm. ^13^C{^1^H} NMR (100.61 MHz, CDCl_3_): *δ* 11.90 and 13.60 (both s, Me), 46.25 and 49.10 (both s, CH_2_), 119.54 (s, C6), 121.49 and 123.55 (both s, C9 and C12), 124.32 (s, C4), 125.97, 126.25, and 127.55 (three s, C5, C10, and C11), 131.11 and 131.57 (both s, C3 and C13), 143.31 (s, C2), 150.94 and 151.32 (both s, C1 and C8), 172.91 (s, C=S), 176.05 (s, C7) ppm. IR (KBr, *ν*/cm^−1^): 431(vw), 675(vw), 694(w), 700(w), 730(vw), 756(m), 768(m), 784(w), 826(vw), 917(w), 951(w), 1017(w), 1083(w), 1099(w), 1148(w), 1185(w), 1227(m), 1249(m), 1262(m), 1272(m), 1294(s), 1312(s), 1361(m), 1380(w), 1419(m), 1434(m), 1446(m), 1457(m), 1486(w), 1529(br, s), 1592(vw), 2869(vw), 2933(w), 2977(w), 3062(w). Anal. Cacld for C_18_H_17_ClN_2_OPdS_2_: C, 44.73; H, 3.55; N, 5.80. Found: C, 44.53; H, 3.75; N, 6.00%.

#### 3.2.5. Solid-Phase Synthesis of [*κ*^3^-*S*,*C*,*N*-(L)Pd(II)Cl] Complexes **2a**,**b**

The corresponding ligand (0.143 mmol) and PdCl_2_(NCPh)_2_ (55 mg, 0.143 mmol) were manually ground in a mortar for 20 min, alternating between periods of grinding with short breaks and scraping the reaction mixture with a spatula. This resulted in an orange (in the case of ligand **1a**) or brown (in the case of ligand **1b**) slightly oily powder. The latter was heated in an open test tube at 100 (**1b**) or 125 °C (**1a**) for 20 min. During this period of time, the initial powder sample rapidly converted to a brown semi-solid, which then slowly solidified and lightened to give a light yellow free-flowing powder. The latter was analyzed by IR spectroscopy (see Figures S25 and S26 in the SI), PXRD (Figures S29–S32 in the SI), and elemental analysis without any workup, which revealed the quantitative formation of the target pincer complexes. Anal. Calcd for C_16_H_13_ClN_2_OPdS_2_ (**2a**): C, 42.21; H, 2.88; N, 6.15. Found: C, 42.30; H, 3.06; N, 6.17%. Anal. Cacld for C_18_H_17_ClN_2_OPdS_2_ (**2b**): C, 44.73; H, 3.55; N, 5.80. Found: C, 44.52; H, 3.68; N, 5.88%.

### 3.3. X-ray Crystallography

Single crystals suitable for X-ray diffraction analysis were obtained by slow diffusion of Et_2_O into an *N*-methyl-2-pyrrolidone solution of complex **2a**. The data were collected at 100 K with a Bruker APEX II CCD diffractometer, using graphite monochromated Mo-Kα radiation (λ = 0.71073 Å). Using Olex2 [47], the structure was solved with the ShelXT structure solution program [48] using Intrinsic Phasing and refined with the XL refinement package [49] using Least Squares minimization against F^2^_hkl_ in anisotropic approximation for non-hydrogen atoms. The positions of hydrogen atoms were calculated, and they were refined in the isotropic approximation within the riding model. The crystal data and structure refinement parameters are given in Table S1 in the SI. CCDC 2311540 contains the supplementary crystallographic data.

### 3.4. Powder X-ray Diffraction Analysis

To measure X-ray diffraction patterns, the dispersions of the powder samples in hexane were deposited on a flat holder made of Si(911) wafer. The measurements were carried out with a D8 Advance diffractometer (Bruker AXS) in the Bragg–Brentano focusing geometry using CuKα radiation; the scan rate was 0.5 deg·min^−1^, and the angular step was 0.02°. The powder patterns were processed with DIFFRACplus EVA [50]. The indexing and Pawley fit of the XRD patterns were carried out using DIFFRAC TOPAS 5.0 software [51].

### 3.5. Bioactivity Studies

The cytotoxicities of ligand **1b** and Pd(II) pincer complexes **2a**,**b**, **V**, and **VI** (X = O) were studied on human colorectal carcinoma (HCT116), breast cancer (MCF7), prostate adenocarcinoma (PC3), chronic myelogenous leukemia (K562 and K562/iS9), multiple plasmacytoma (AMO1), and acute lymphoblastic leukemia (H9) cell lines, as well as human embryonic kidney (HEK293) and mammary epithelial (HBL100 and HBL100/Dox) cells used as representatives of non-cancerous cell lines. All the cell lines were obtained from American Type Culture Collection (ATCC). The tested compounds were initially dissolved in DMSO. Cisplatin (in the initial form of an infusion concentrate in natural saline solution) from a commercial source was used as a positive control. The detailed procedure is described in Ref. [52].

The apoptosis-inducing ability of complex **2a** was explored on K562 and K562/iS9 chronic myelogenous leukemia cells according to the published procedure [52] using 1 μM concentration of the mentioned palladacycle.

## 4. Conclusions

In summary, this report demonstrated the successful use of a new class of benzothiazole–thiocarbamate pincer ligands in the creation of new promising palladium-based cytotoxic agents. Among the palladacycles explored, the dimethylthiocarbamoyl-substituted derivative exhibited the highest anticancer potential, combining prominent antiproliferative activity with remarkable apoptosis induction ability. Another valuable finding of this work is that this type of potential metal-based chemotherapeutics can be efficiently produced under solvent-free conditions in full accordance with the modern concept of green chemistry.

## Figures and Tables

**Table 1:** Cytotoxicity of the benzothiazole-appended Pd(II) pincer complexes against some human cell lines.

Cell Lines	IC_50_ ± SD ^1^, μM			
	2a	2b	VI (X = O)	Cisplatin
Solid cancer cell lines				
HCT116	1.3 ± 0.2	12.7 ± 1.5	10.3 ± 0.8	18.0 ± 2.0
MCF7	3.3 ± 0.4	13.5 ± 2.1	17.0 ± 0.5	25.0 ± 4.0
PC3	1.1 ± 0.3	8.5 ± 2.5	6.6 ± 0.6	16.0 ± 3.0
Hematopoietic cancer cell lines				
K562	0.9 ± 0.2	2.9 ± 0.4	1.9 ± 0.3	15.5 ± 0.5
K562/iS9	0.9 ± 0.1	2.6 ± 0.8	2.5 ± 0.4	16.0 ± 2.0
AMO1	0.3 ± 0.1	4.4 ± 0.8	3.4 ± 0.5	3.2 ± 0.6
H9	1.3 ± 0.1	2.1 ± 0.9	1.7 ± 0.3	3.0 ± 1.0
Non-cancerous cell lines				
HEK293	2.5 ± 0.2	9.5 ± 0.7	13.0 ± 0.5	12.5 ± 1.5
HBL100	3.2 ± 0.5	10.2 ± 3.3	9.4 ± 0.9	14.6 ± 3.6
HBL100/dox	4.4 ± 0.8	16.3 ± 3.8	28.6 ± 3.8	23.6 ± 3.6

^1^ SD is the standard deviation of the value.

## Data Availability

The data presented in this study are available in the article and supplementary materials.

## Supplementary Materials

The following supporting information can be downloaded at: https://www.mdpi.com/article/10.3390/ijms242417331/s1.
