# Supplementary material for: Unsymmetrical Pd(II) Pincer Complexes with Benzothiazole and Thiocarbamate Flanking Units: Expedient Solvent-Free Synthesis and Anticancer Potential
Source: Int J Mol Sci. 2023 Dec 10;24(24):17331. doi: 10.3390/ijms242417331 (PMC10744248; doi:10.3390/ijms242417331)
Supplement: Supplementary file 1 [file ijms-24-17331-s001.zip › ijms-2730193-supplementary.pdf]

# Supplementary materials

# Unsymmetrical Pd(II) pincer complexes with benzothiazole and thiocarbamate flanking units: expedient solvent-free synthesis and anticancer potential

Vladimir A. Kozlov,[a] Diana V. Aleksanyan,[a,b]* Svetlana G. Churusova,[a] Aleksandr A. Spiridonov,[a] Ekaterina Yu. Rybalkina,[c] Evgenii I. Gutsul,[a] Svetlana A. Aksenova,[a] Alexander A. Korlyukov,[a] Alexander S. Peregudov,[a] and Zinaida S. Klemenkova[a]

[a] *A. N. Nesmeyanov Institute of Organoelement Compounds, Russian Academy of Sciences, ul. Vavilova 28, str. 1, Moscow, 119334 Russia*

[b] *Scientific Laboratory "Advanced Composite Materials and Technologies",Plekhanov Russian University of Economics, Stremyannyi per. 36, Moscow, 117997 Russia*

[c] *N. N. Blokhin National Medical Research Center of Oncology of the Ministry of Health of the Russian Federation, Kashirskoe shosse 23, Moscow, 115478 Russia*

*corresponding author: aleksanyan.diana@ineos.ac.ru

## Table of contents

| | Page |
|---|---|
| **Fig. S1.** $^1$H NMR spectrum of ligand **1a** (500.13 MHz, $(CD_3)_2SO$) | S3 |
| **Fig. S2.** $^{13}C\{^1H\}$ NMR spectrum of ligand **1a** (125.76 MHz, $(CD_3)_2SO$) | S4 |
| **Fig. S3.** $^1$H–$^1$H COSY spectrum of ligand **1a** (500.13 MHz, $(CD_3)_2SO$) | S5 |
| **Fig. S4.** Extended fragment of the $^1$H–$^1$H COSY spectrum of ligand **1a** (500.13 MHz, $(CD_3)_2SO$) | S6 |
| **Fig. S5.** HSQC spectrum of ligand **1a** ($(CD_3)_2SO$) | S7 |
| **Fig. S6.** Extended fragment of the HSQC spectrum of ligand **1a** ($(CD_3)_2SO$) | S8 |
| **Fig. S7.** $^1$H–$^{13}$C HMBC spectrum of ligand **1a** ($(CD_3)_2SO$) | S9 |
| **Fig. S8.** Extended fragments of the $^1$H–$^{13}$C HMBC spectrum of ligand **1a** ($(CD_3)_2SO$) | S10 |
| **Fig. S9.** $^1$H NMR spectrum of ligand **1b** (400.13 MHz, $CDCl_3$) | S11 |
| **Fig. S10.** $^{13}C\{^1H\}$ NMR spectrum of ligand **1b** (100.61 MHz, $CDCl_3$) | S12 |
| **Fig. S11.** $^1$H NMR spectrum of complex **2a** (500.13 MHz, $(CD_3)_2SO$) | S13 |
| **Fig. S12.** $^{13}C\{^1H\}$ NMR spectrum of complex **2a** (125.76 MHz, $(CD_3)_2SO$) | S14 |
| **Fig. S13.** $^1$H–$^1$H COSY spectrum of complex **2a** (500.13 MHz, $(CD_3)_2SO$) | S15 |
| **Fig. S14.** Extended fragments of the $^1$H–$^1$H COSY spectrum of complex **2a** (500.13 MHz, $(CD_3)_2SO$) | S16 |
| **Fig. S15.** HSQC spectrum of complex **2a** ($(CD_3)_2SO$) | S17 |
| **Fig. S16.** Extended fragment of the HSQC spectrum of complex **2a** ($(CD_3)_2SO$) | S18 |
| **Fig. S17.** $^1$H–$^{13}$C HMBC spectrum of complex **2a** ($(CD_3)_2SO$) | S19 |

**Fig. S18.** Extended fragments of the $^{1}H–^{13}C$ HMBC spectrum of complex **2a** ($(CD_3)_2SO$) S20

**Fig. S19.** $^{1}H$ NMR spectrum of complex **2b** (400.13 MHz, $CDCl_3$) S21

**Fig. S20.** $^{13}C\{^{1}H\}$ NMR spectrum of complex **2b** (100.61 MHz, $CDCl_3$) S22

**Fig. S21.** IR spectrum of ligand **1a** S23

**Fig. S22.** IR spectrum of ligand **1b** S24

**Fig. S23.** IR spectrum of complex **2a** S25

**Fig. S24.** IR spectrum of complex **2b** S26

**Fig. S25.** IR spectrum of a light yellow solid obtained after heating of the ground mixture of ligand **1a** and $PdCl_2(NCPh)_2$ S27

**Fig. S26.** IR spectrum of a light yellow solid obtained after heating of the ground mixture of ligand **1b** and $PdCl_2(NCPh)_2$ S28

**Fig. S27.** IR spectrum of an orange slightly oily powder obtained by grinding ligand **1a** with $PdCl_2(NCPh)_2$ S29

**Fig. S28.** IR spectrum of a brown slightly oily powder obtained by grinding ligand **1b** with $PdCl_2(NCPh)_2$ S30

**Fig. S29.** XRD patterns for an oily solid residue obtained by grinding ligand **1a** with $PdCl_2(NCPh)_2$ in a mortar (red) and a light yellow powder obtained after its heating (magenta), as well as an authentic sample of pincer complex **2a** derived from the conventional solution-based synthesis (blue) S31

**Fig. S30.** XRD patterns for an oily solid residue obtained by grinding ligand **1b** with $PdCl_2(NCPh)_2$ in a mortar (red) and a light yellow powder obtained after its heating (magenta), as well as an authentic sample of pincer complex **2b** derived from the conventional solution-based synthesis (blue) S31

**Fig. S31.** XRD pattern and the results of the Pawley fit for a solid sample obtained by heating of the ground mixture of ligand **1a** and $PdCl_2(NCPh)_2$. The experimental, calculated, and difference curves are shown by blue, red and grey colors, respectively S32

**Fig. S32.** XRD pattern and the results of the Pawley fit for a solid sample obtained by heating of the ground mixture of ligand **1b** and $PdCl_2(NCPh)_2$. The experimental, calculated, and difference curves are shown by blue, red and grey colors, respectively S32

**Fig. S33.** UV-Vis spectra of complex **2b** in DMSO (***a***), DMSO–deionized water (1:1) (***b***), and DMSO–PBS (1:1) (***c***) registered immediately after dissolution, in one or two days S33

**Fig. S34.** UV-Vis spectra of complex **2b** in DMSO, DMSO–deionized water (1:1), and DMSO–PBS (1:1) registered immediately after dissolution (***a***), in one (***b***) or two (***c***) days S34

**Fig. S35.** UV-Vis spectra of complex **2b** in DMSO and the precipitate from DMSO–PBS (1:1, 2 days) in DMSO registered immediately after dissolution S35

**Table S1.** Crystal data and structure refinement parameters for complex **2a** S36

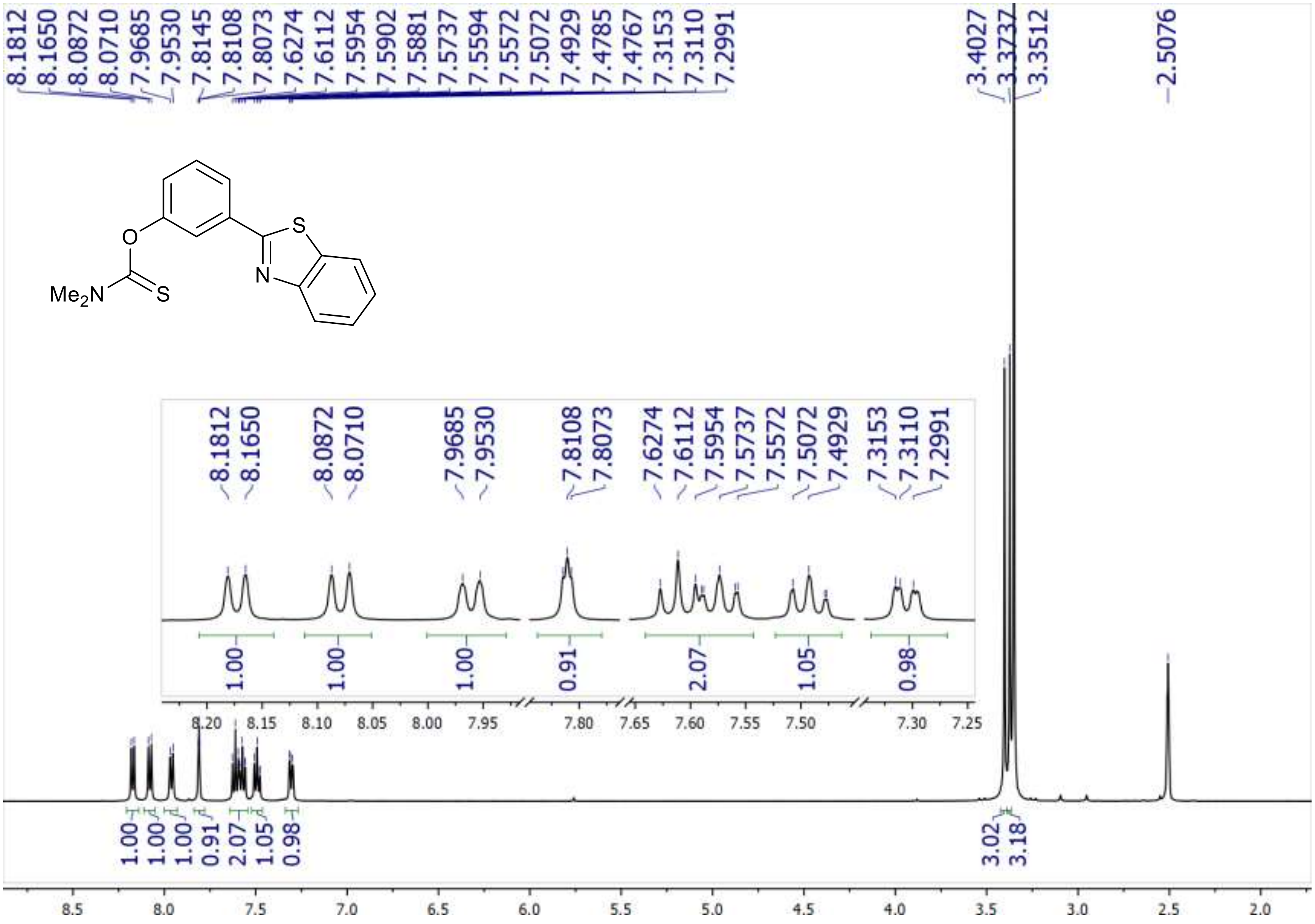


**Figure S1.** $^1$H NMR spectrum of ligand **1a** (500.13 MHz, $(CD_3)_2SO$)

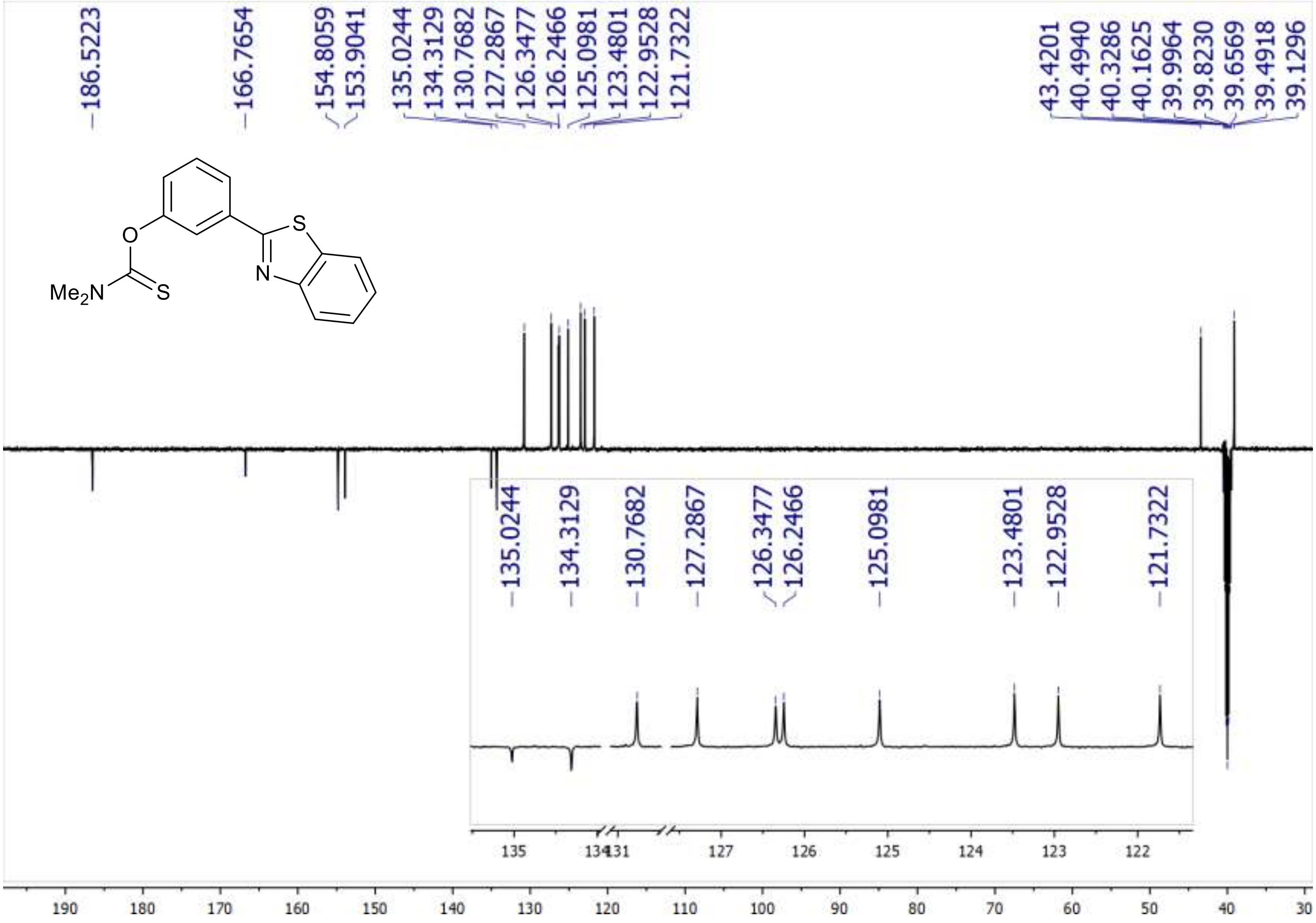


**Figure S2.** $^{13}C\{^{1}H\}$ NMR spectrum of ligand **1a** (125.76 MHz, $(CD_3)_2SO$)

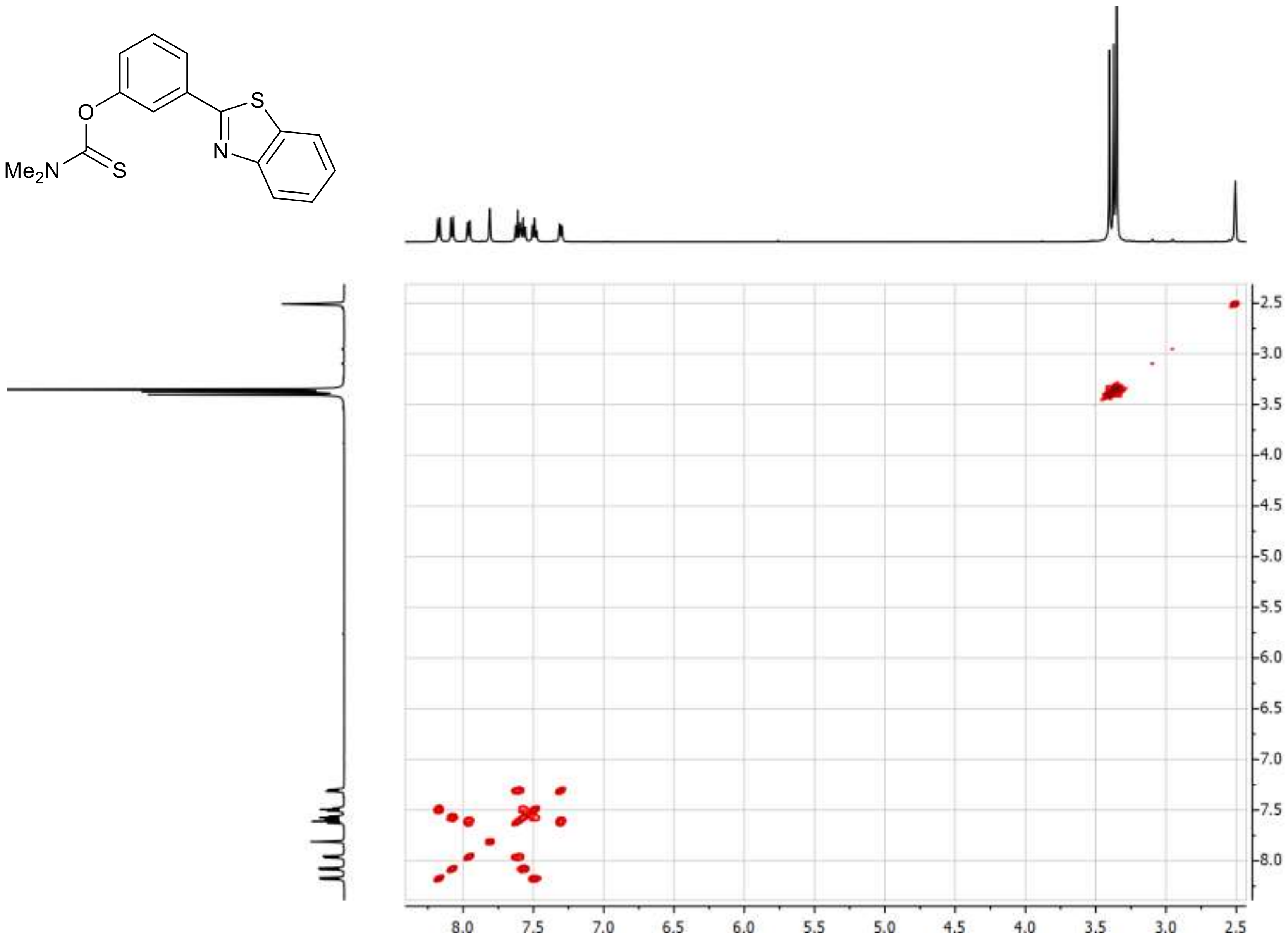


**Figure S3.** $^1H$–$^1H$ COSY spectrum of ligand **1a** (500.13 MHz, $(CD_3)_2SO$)

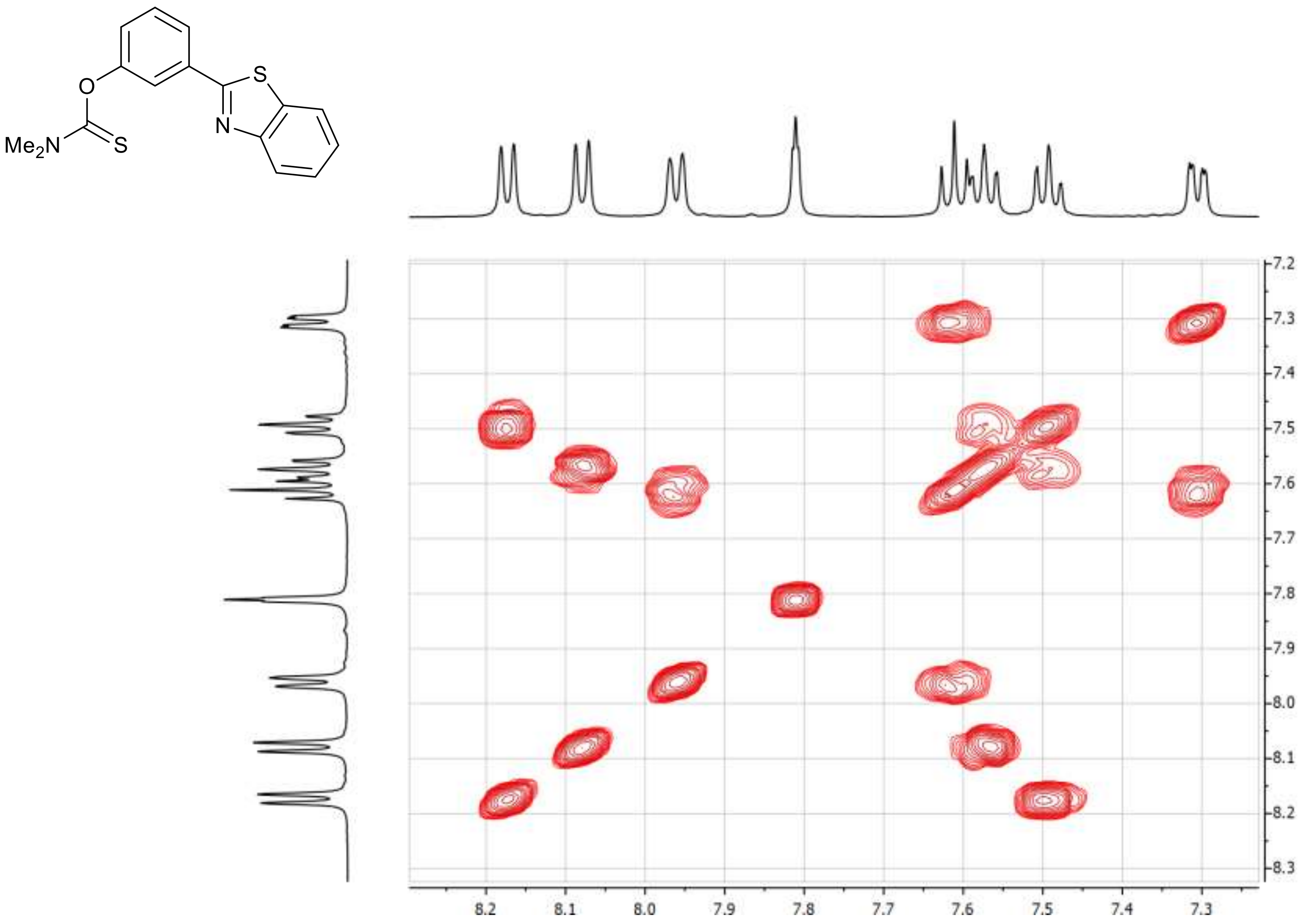


**Figure S4.** Extended fragment of the $^{1}H$−$^{1}H$ COSY spectrum of ligand **1a** (500.13 MHz, $(CD_3)_2SO$)

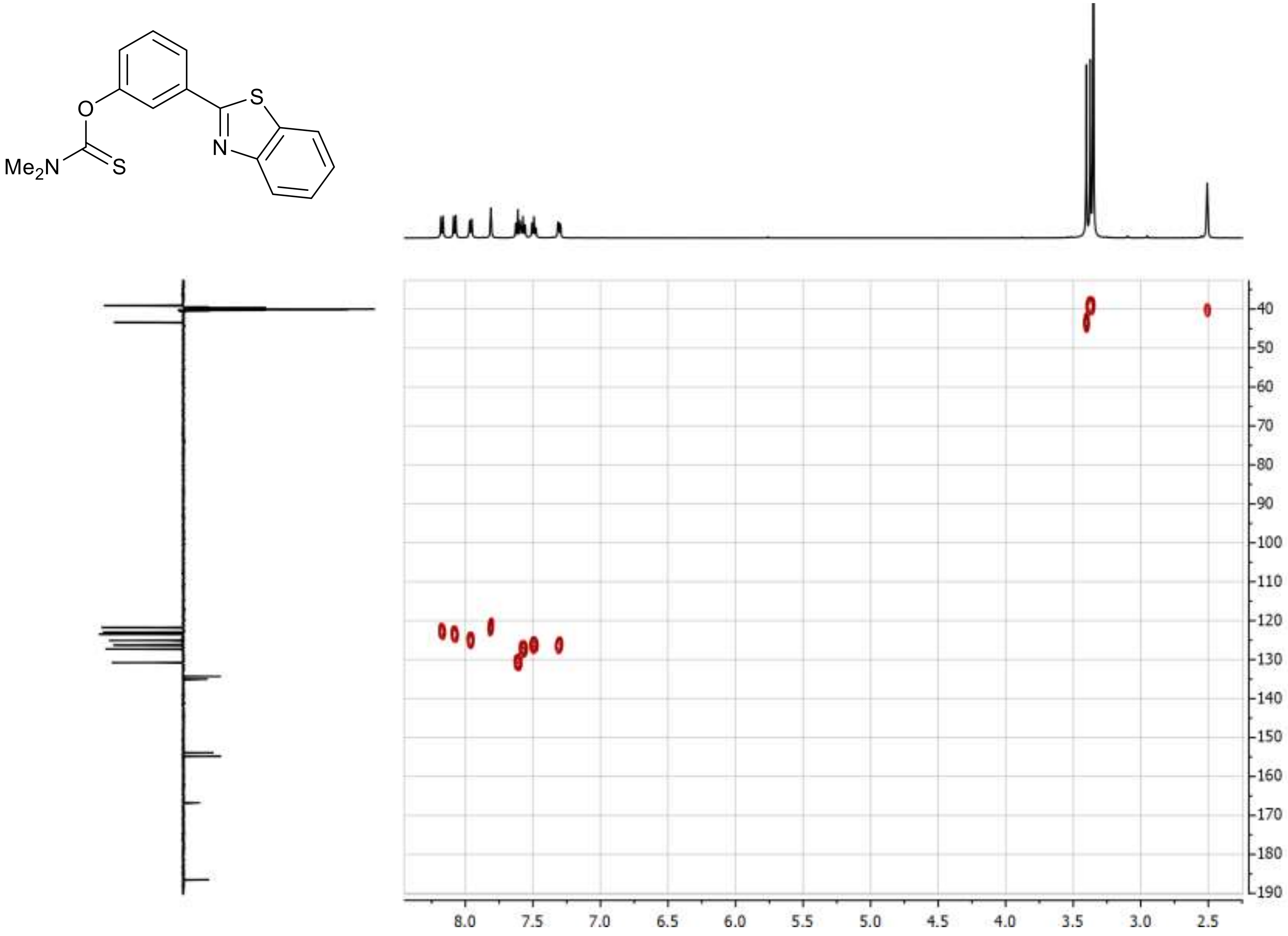


**Figure S5.** HSQC spectrum of ligand **1a** ($(CD_3)_2SO$)

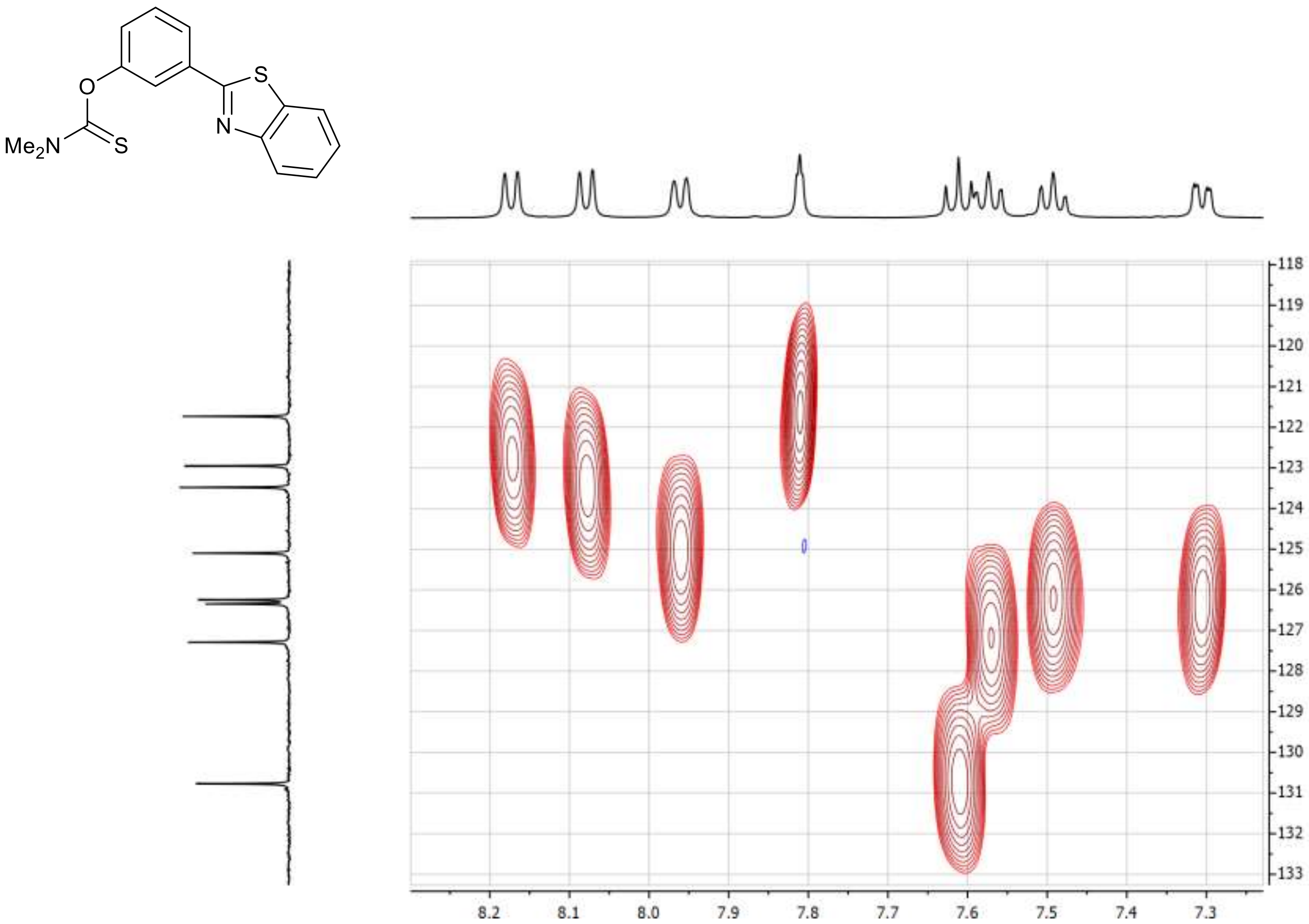


**Figure S6.** Extended fragment of the HSQC spectrum of ligand **1a** ($(CD_3)_2SO$)

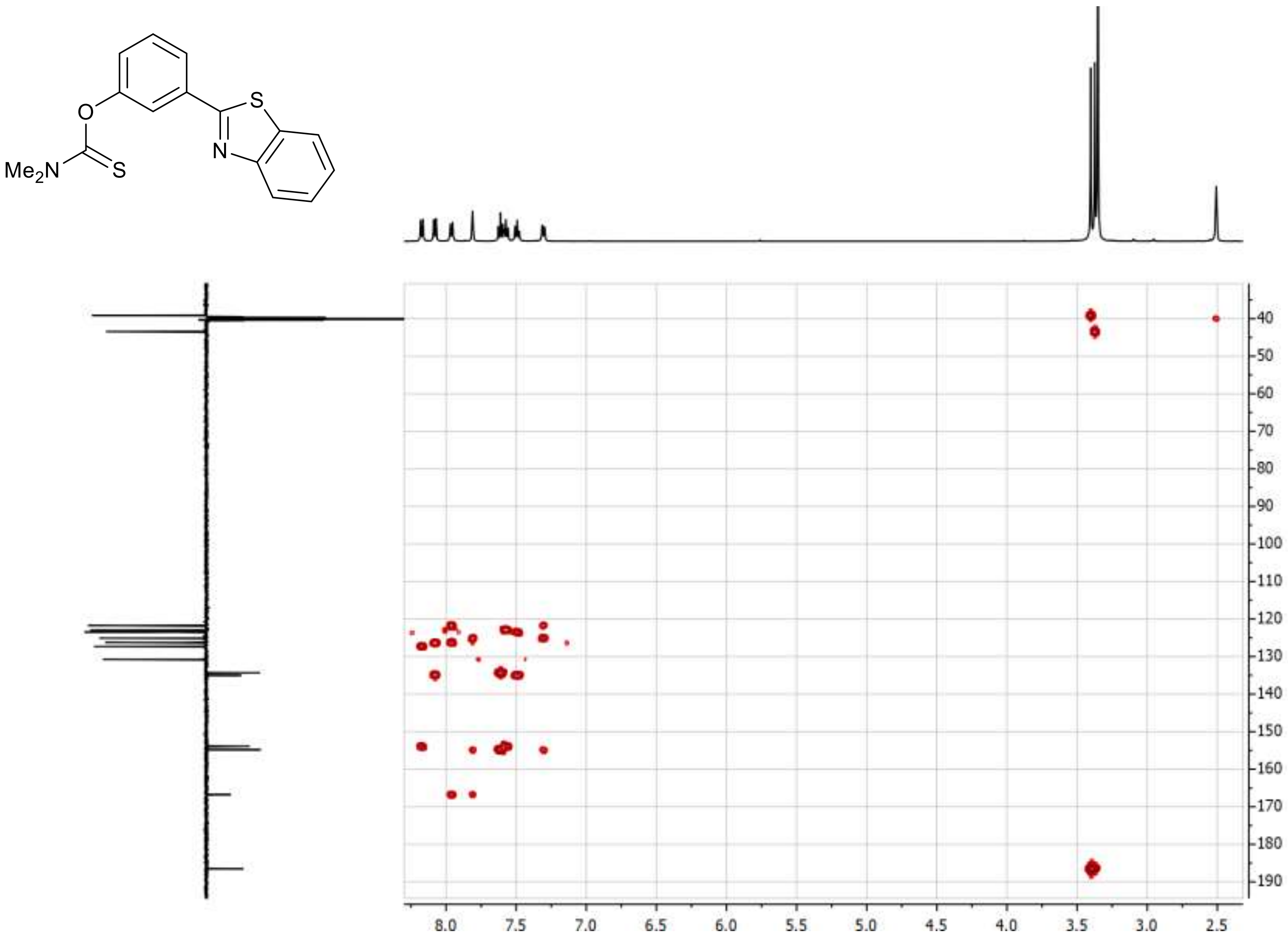


**Figure S7.** $^{1}H-^{13}C$ HMBC spectrum of ligand **1a** ($(CD_3)_2SO$)

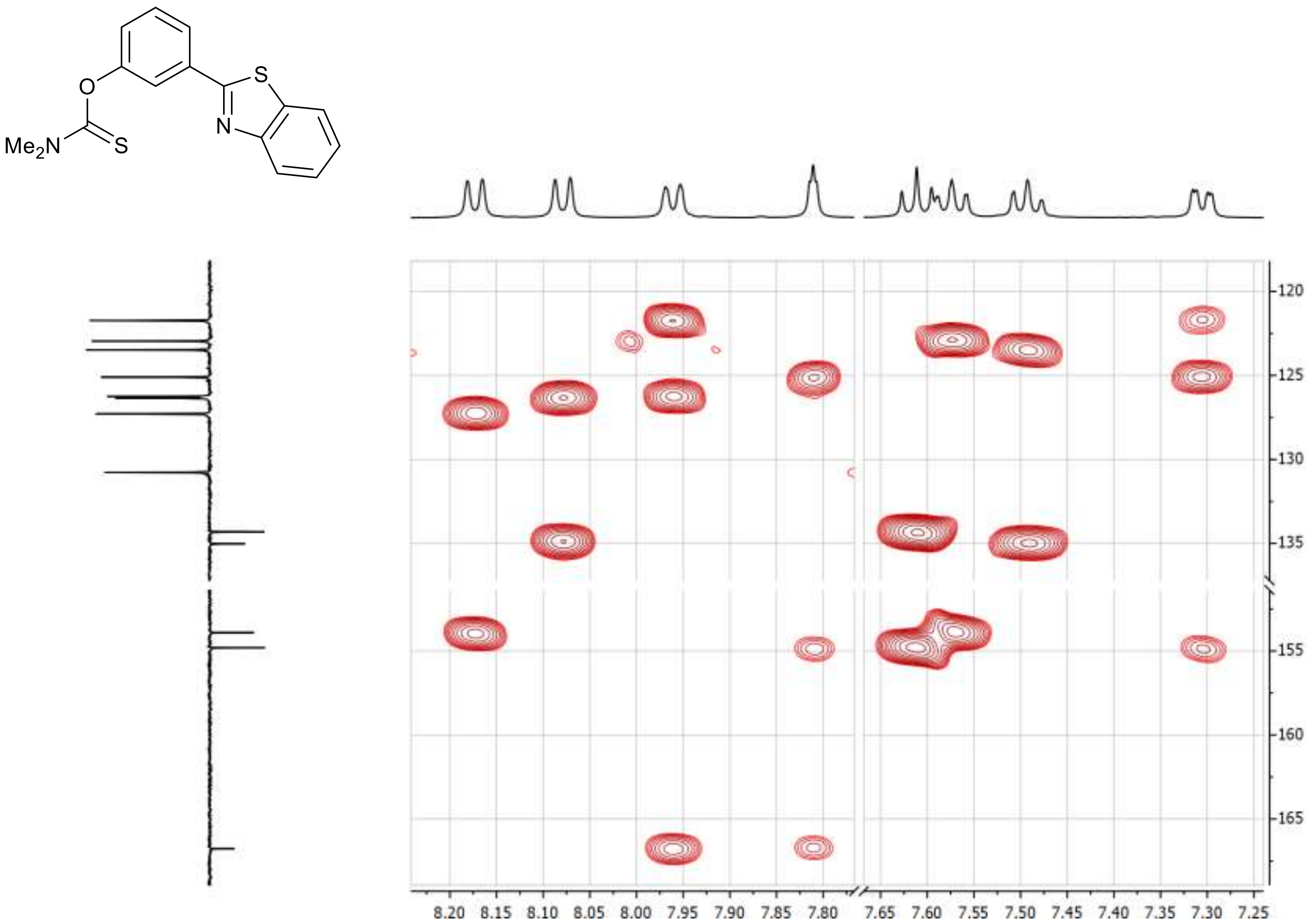


**Figure S8.** Extended fragments of the $^{1}$H−$^{13}$C HMBC spectrum of ligand**1a** ($(CD_3)_2SO$)

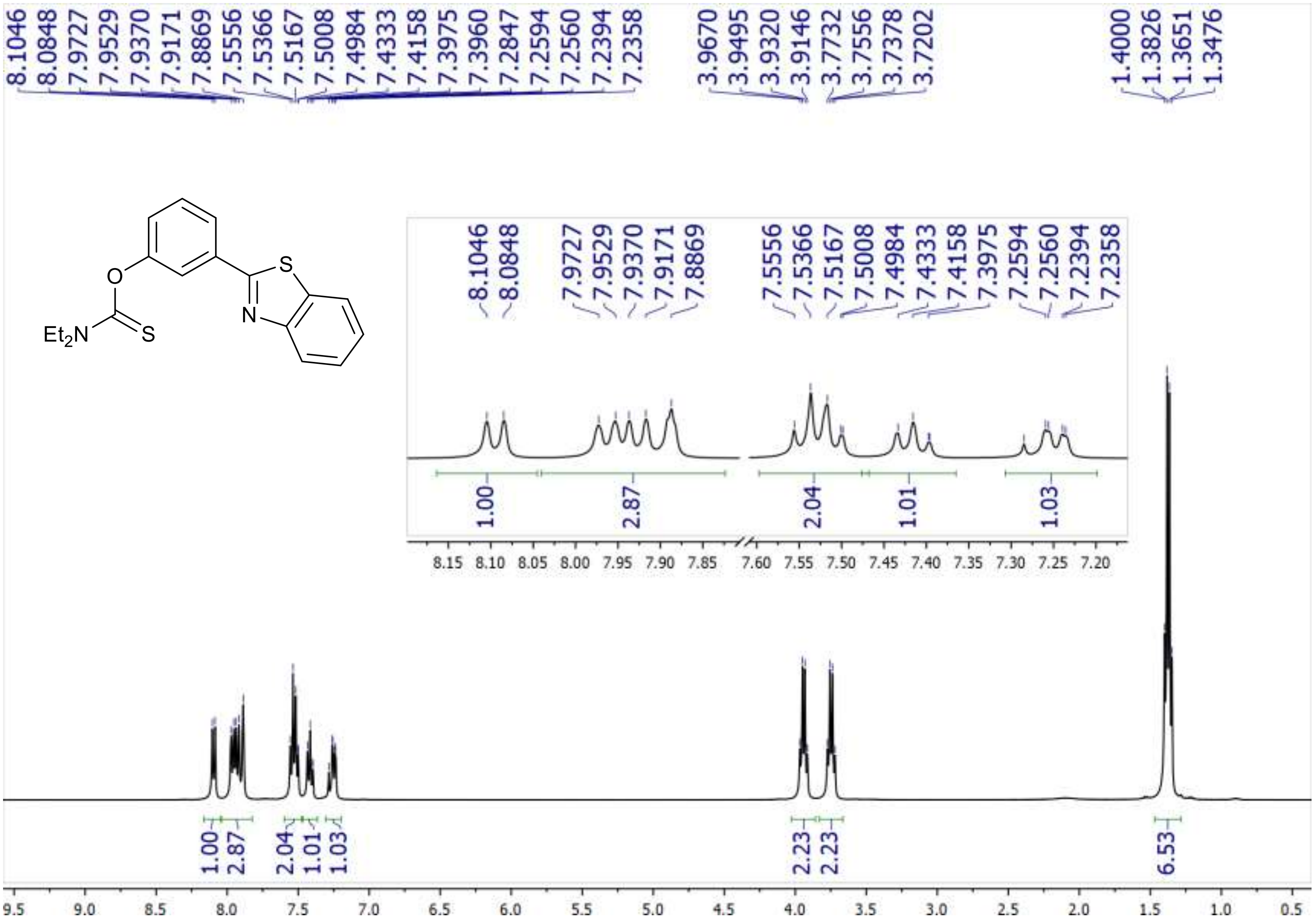


**Figure S9.** $^1$H NMR spectrum of ligand **1b** (400.13 MHz, $CDCl_3$)

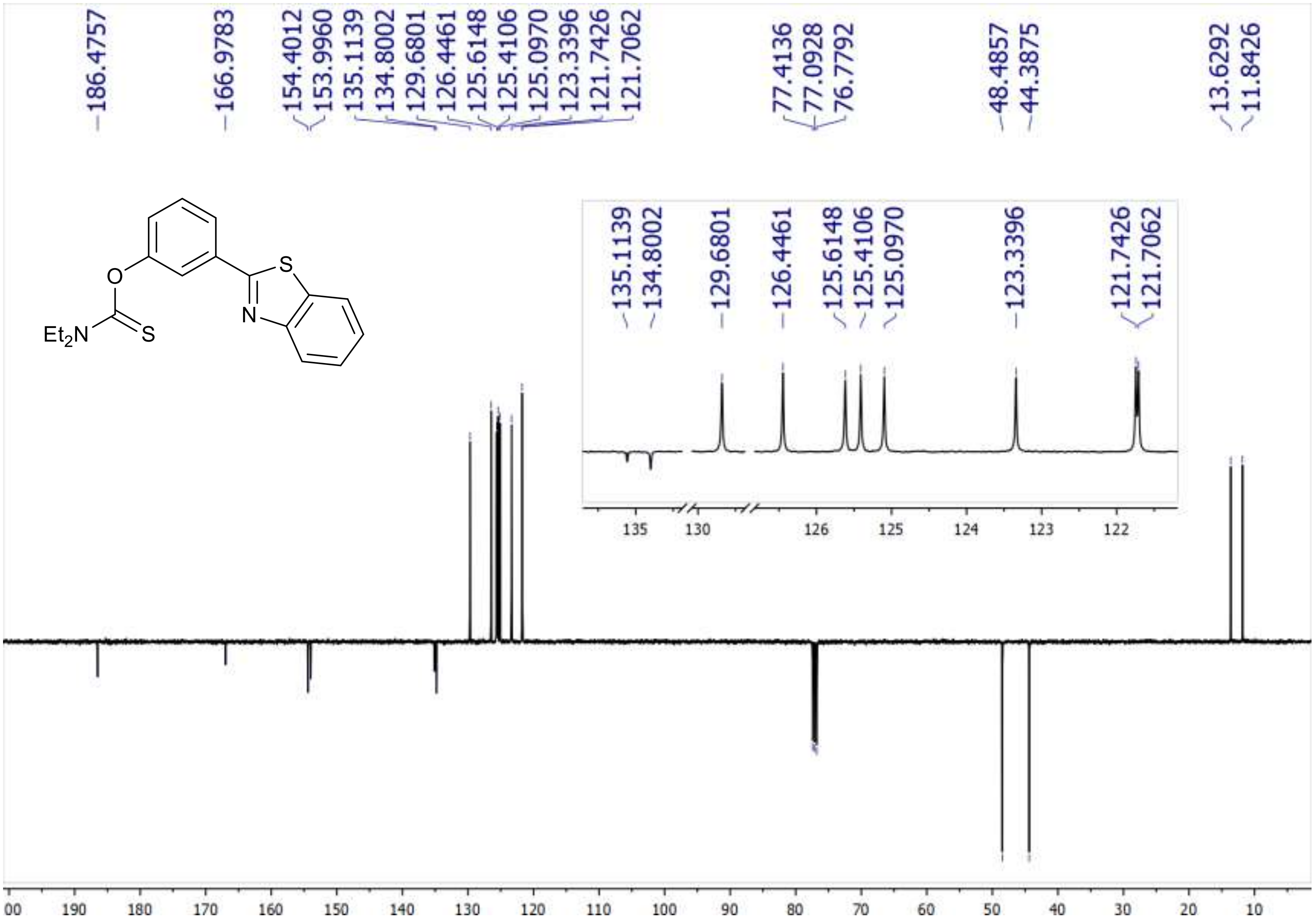


**Figure S10.** $^{13}C\{^1H\}$ NMR spectrum of ligand **1b** (100.61 MHz, $CDCl_3$)

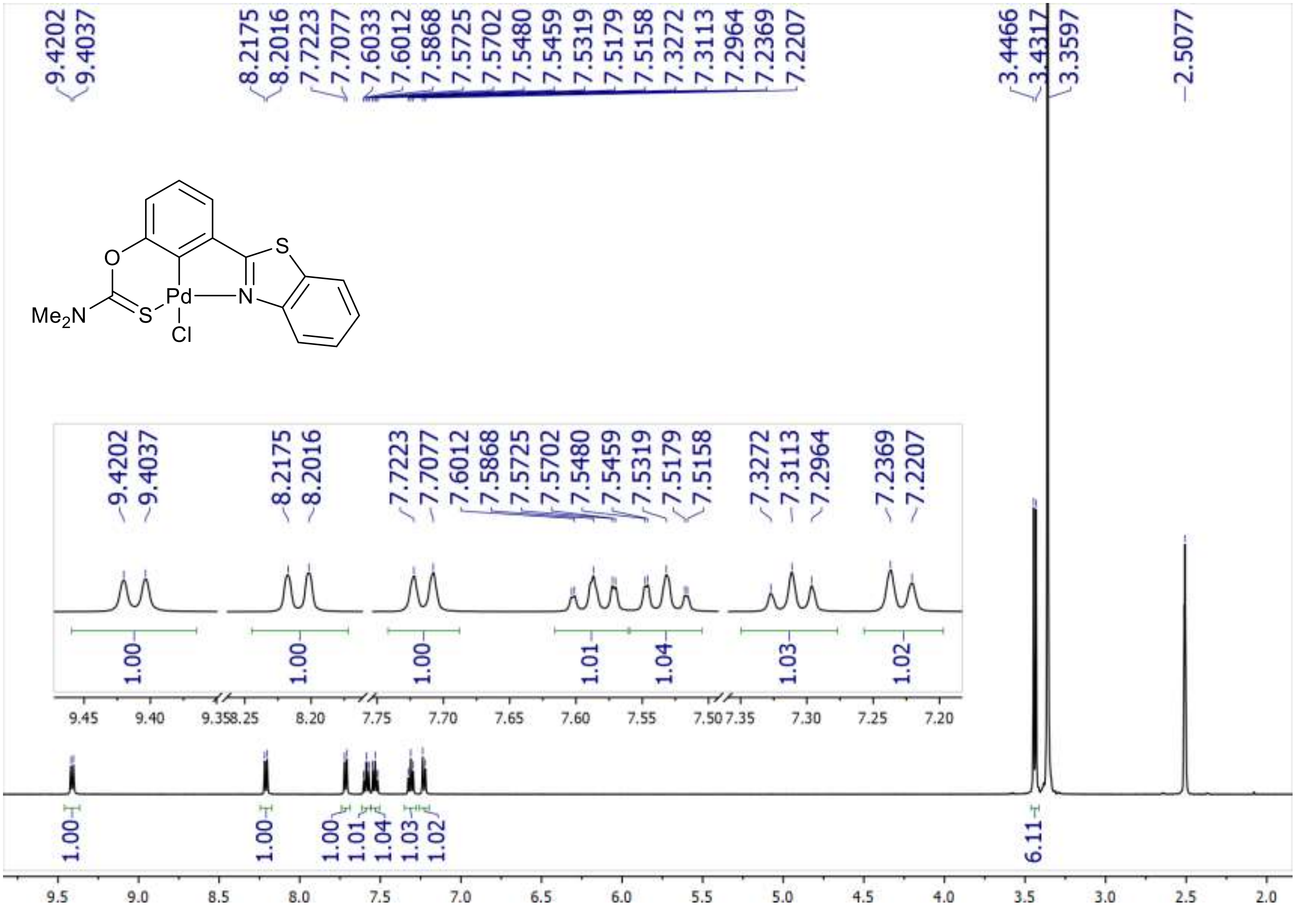


**Figure S11.** $^1$H NMR spectrum of complex **2a** (500.13 MHz, $(CD_3)_2SO$)

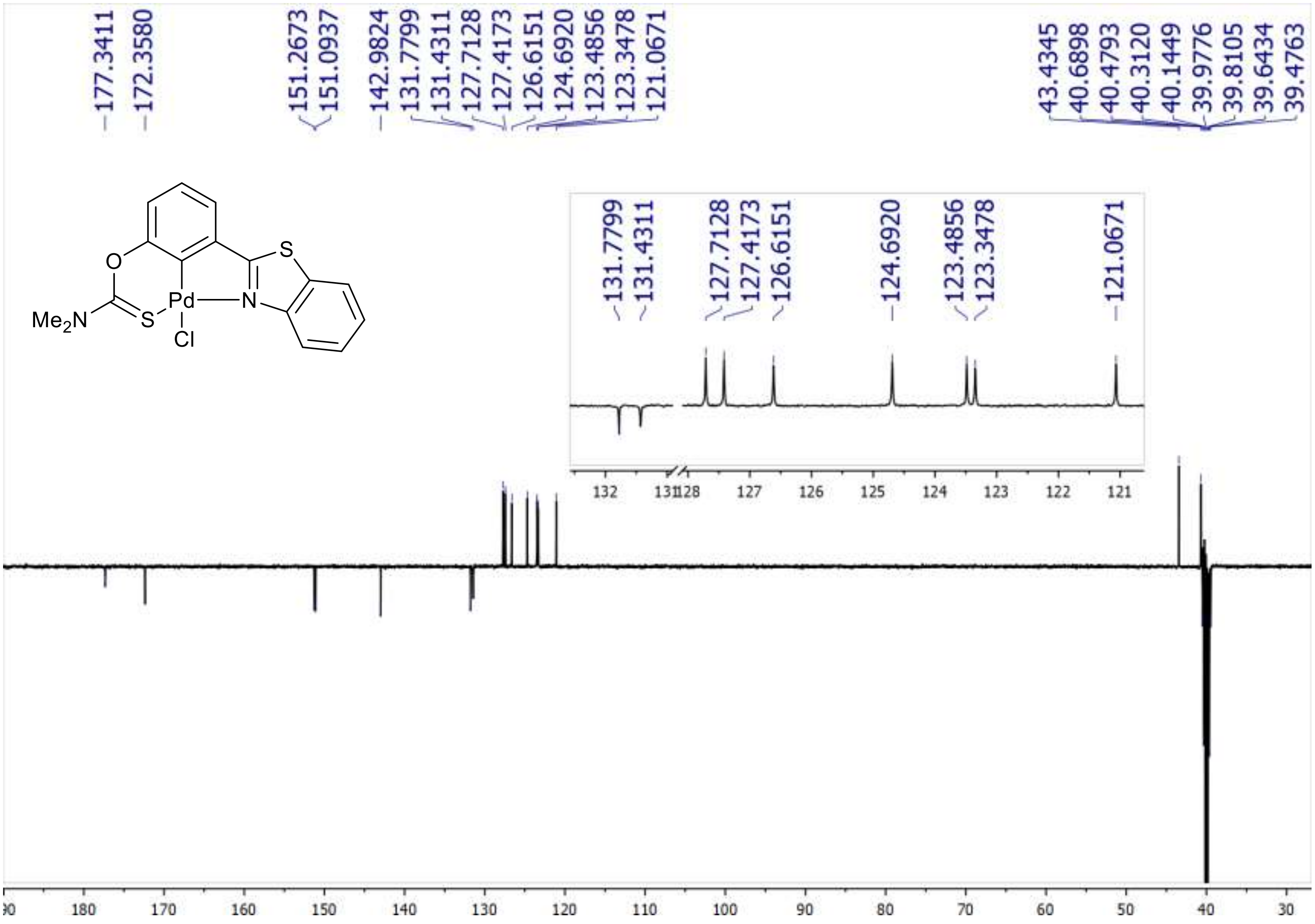


**Figure S12.** $^{13}C\{^1H\}$ NMR spectrum of complex **2a** (125.76 MHz, $(CD_3)_2SO$)

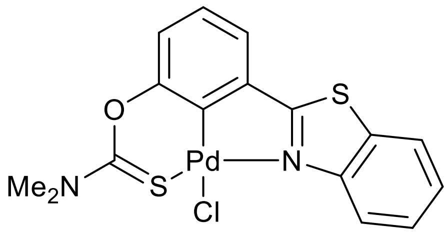


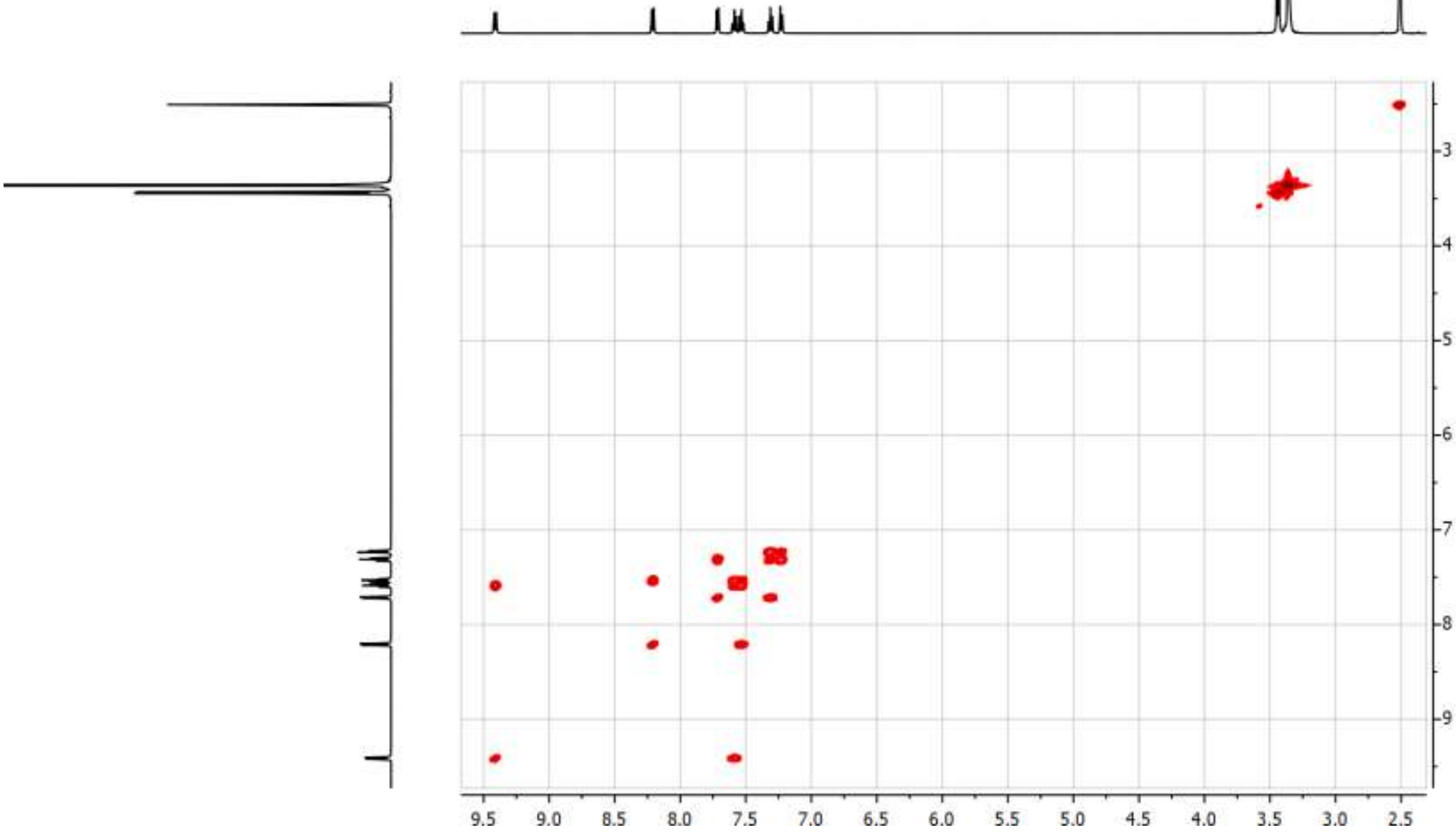

**Figure S13.** $^{1}H-^{1}H$ COSY spectrum of complex **2a** (500.13 MHz, $(CD_3)_2SO$)

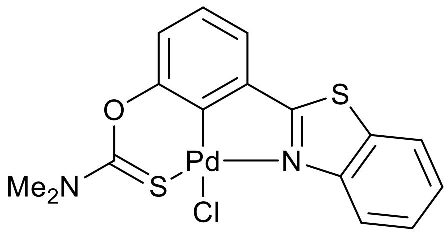


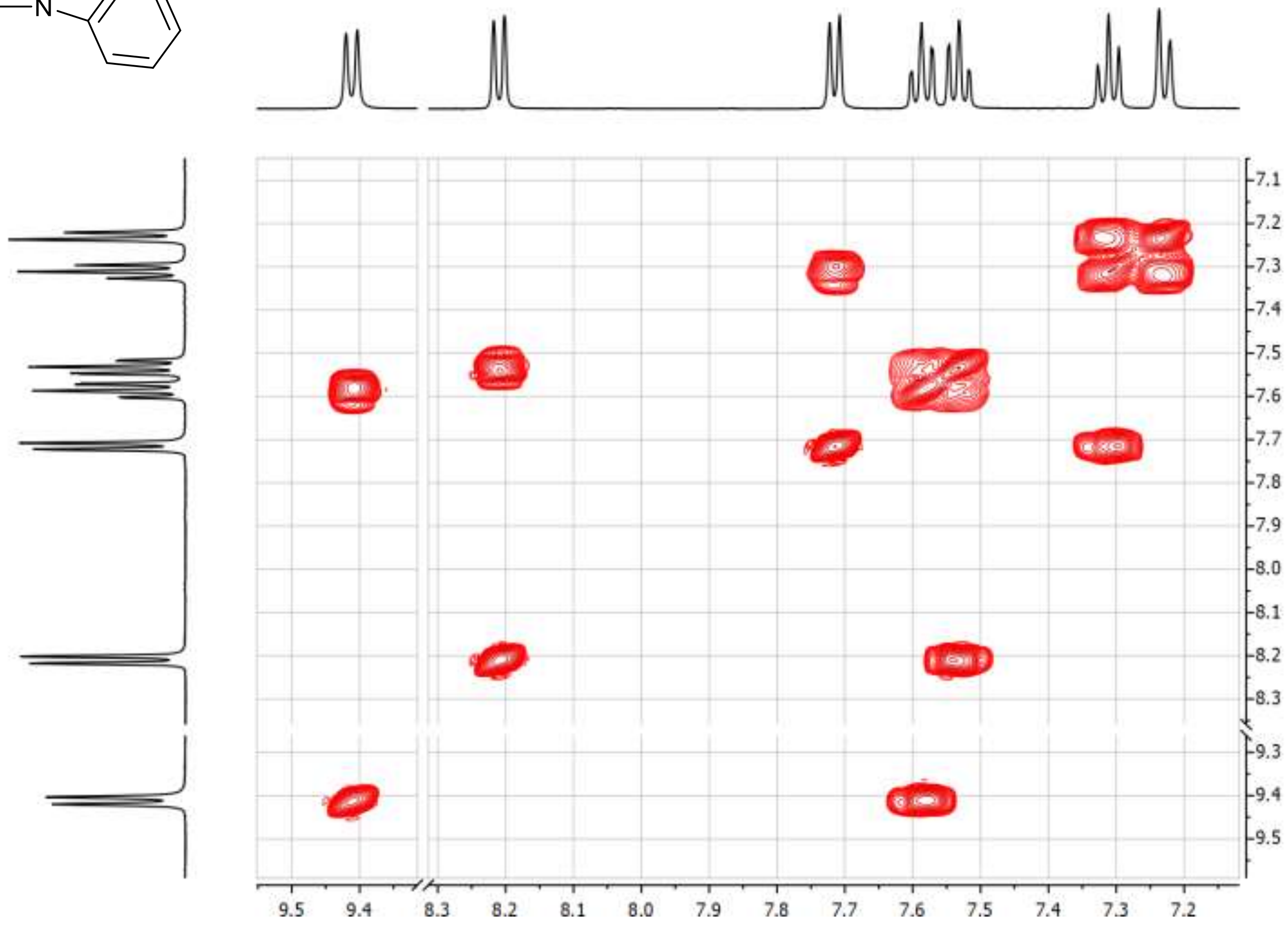

**Figure S14.** Extended fragments of the $^{1}H-^{1}H$ COSY spectrum of complex **2a** (500.13 MHz, $(CD_3)_2SO$)

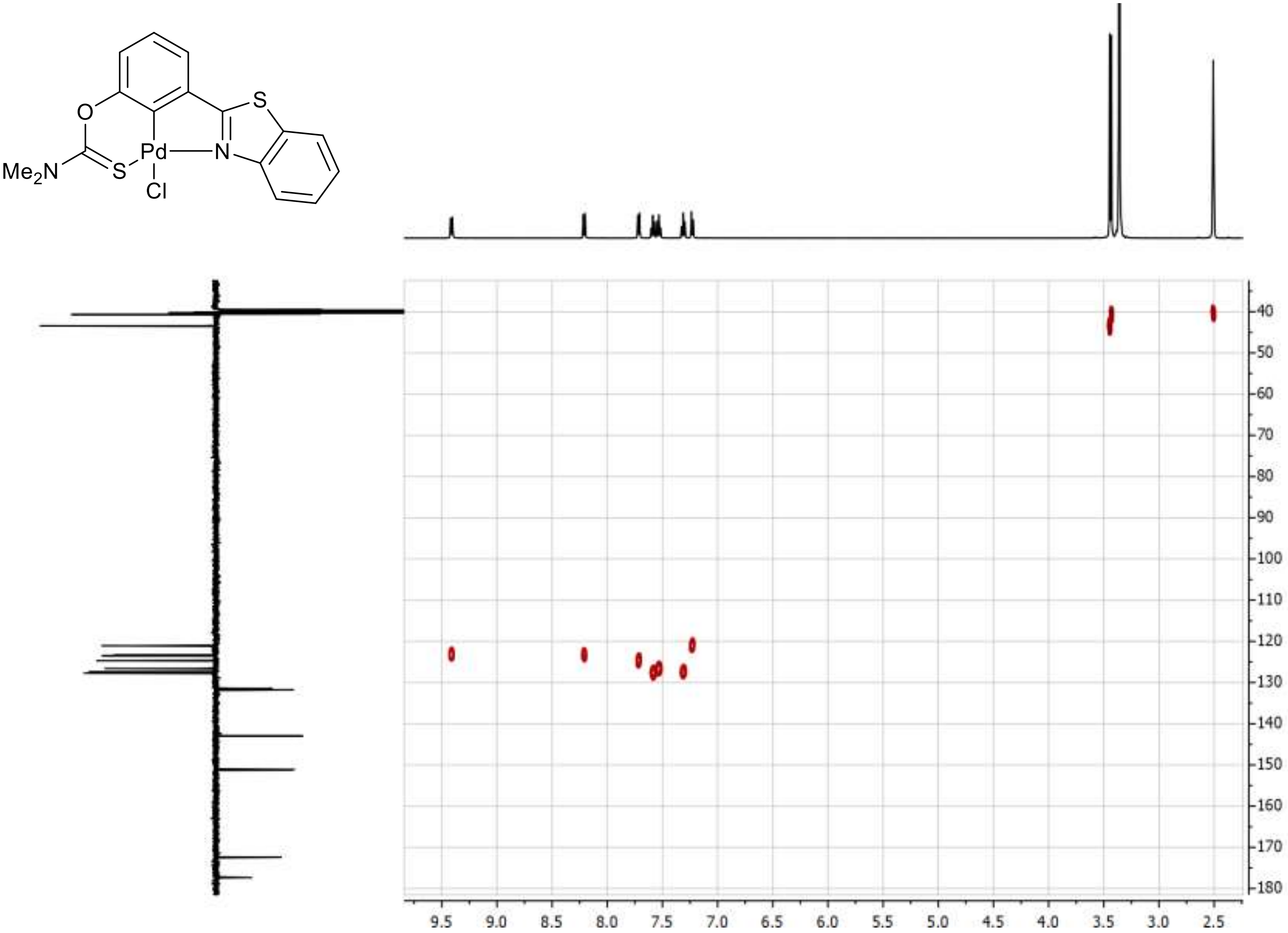


**Figure S15.** HSQC spectrum of complex **2a** ($(CD_3)_2SO$)

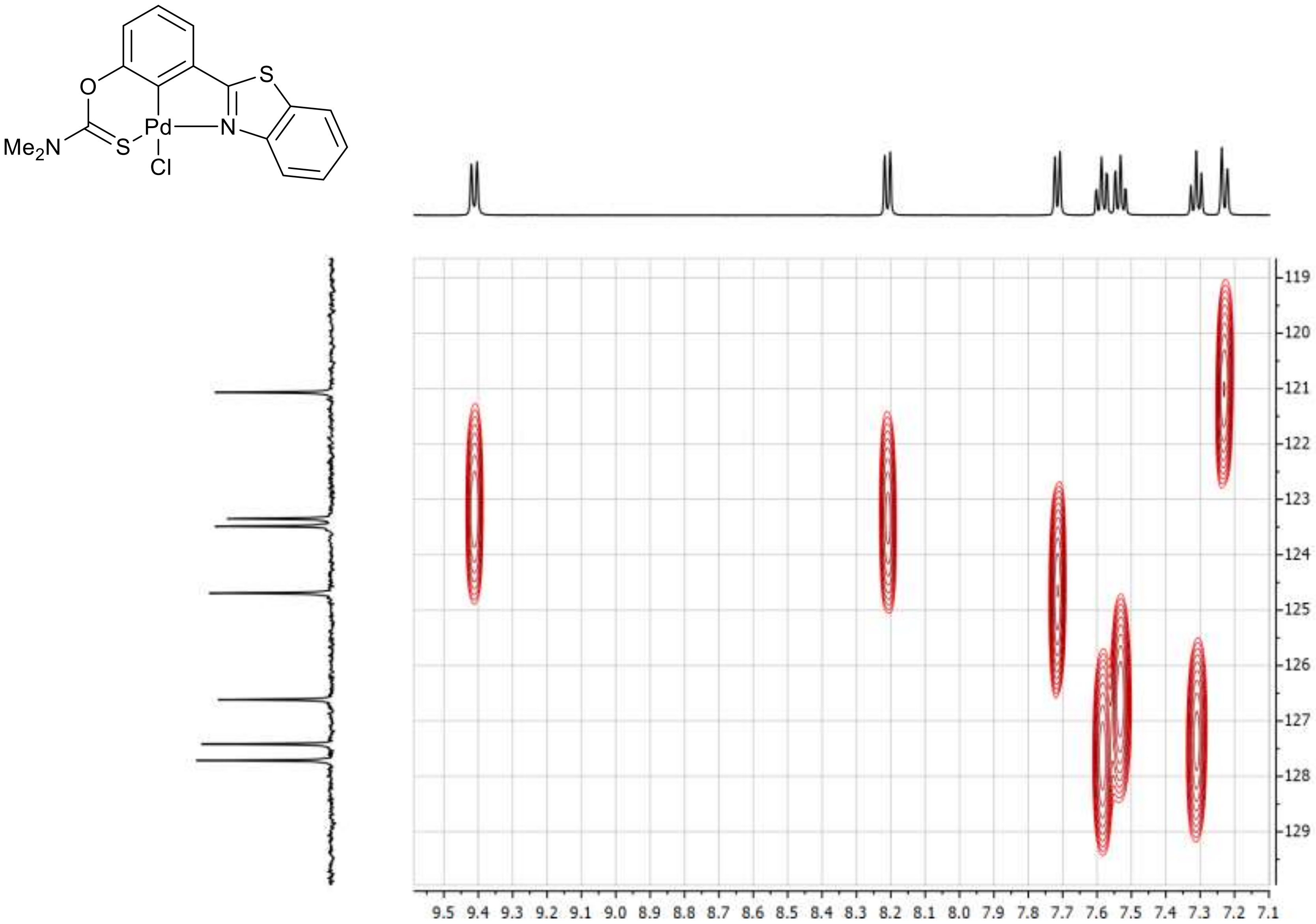


**Figure S16.** Extended fragment of the HSQC spectrum of complex **2a** ($(CD_3)_2SO$)

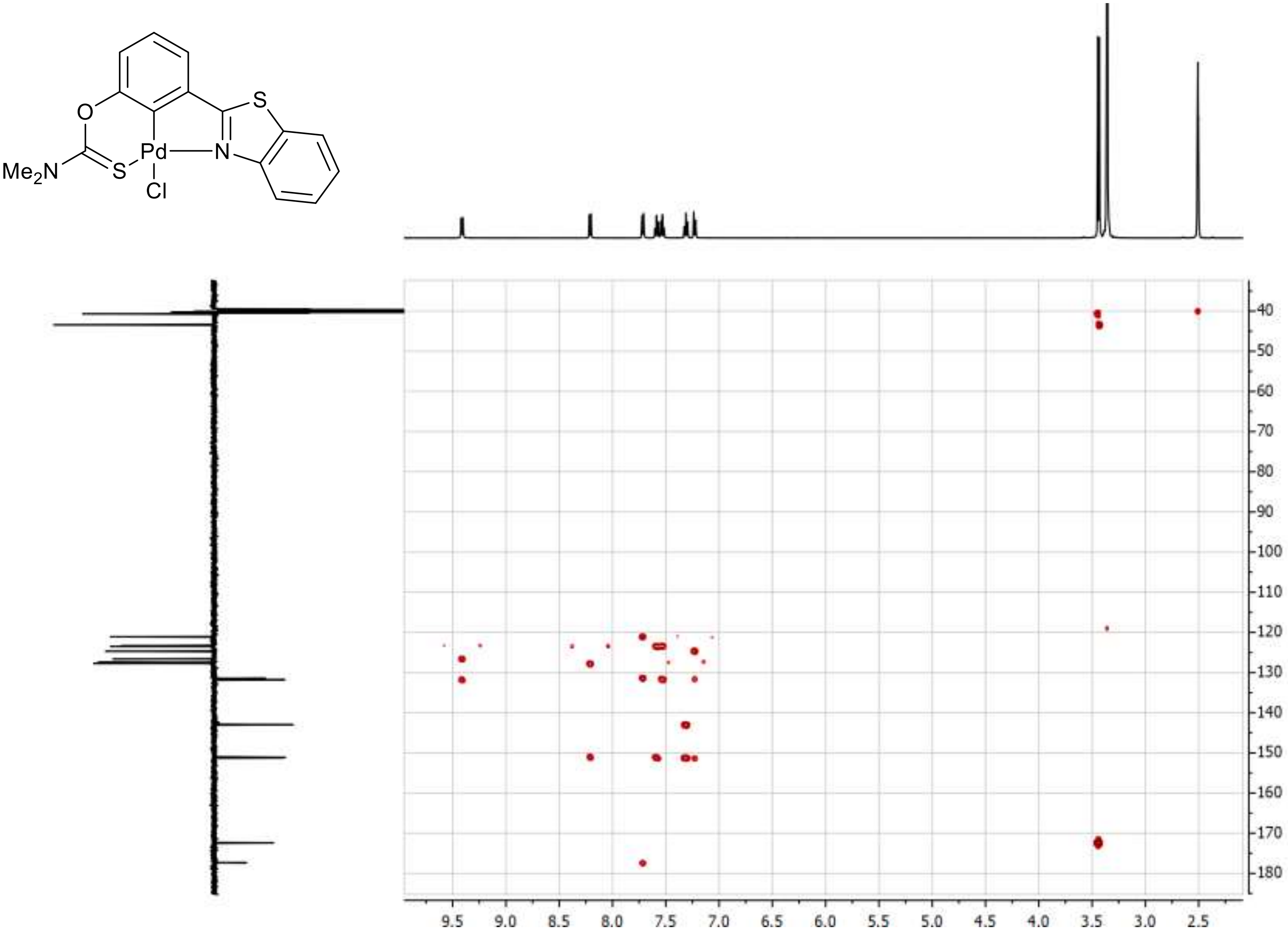


**Figure S17.** $^{1}H-^{13}C$ HMBC spectrum of complex**2a** ($(CD_3)_2SO$)

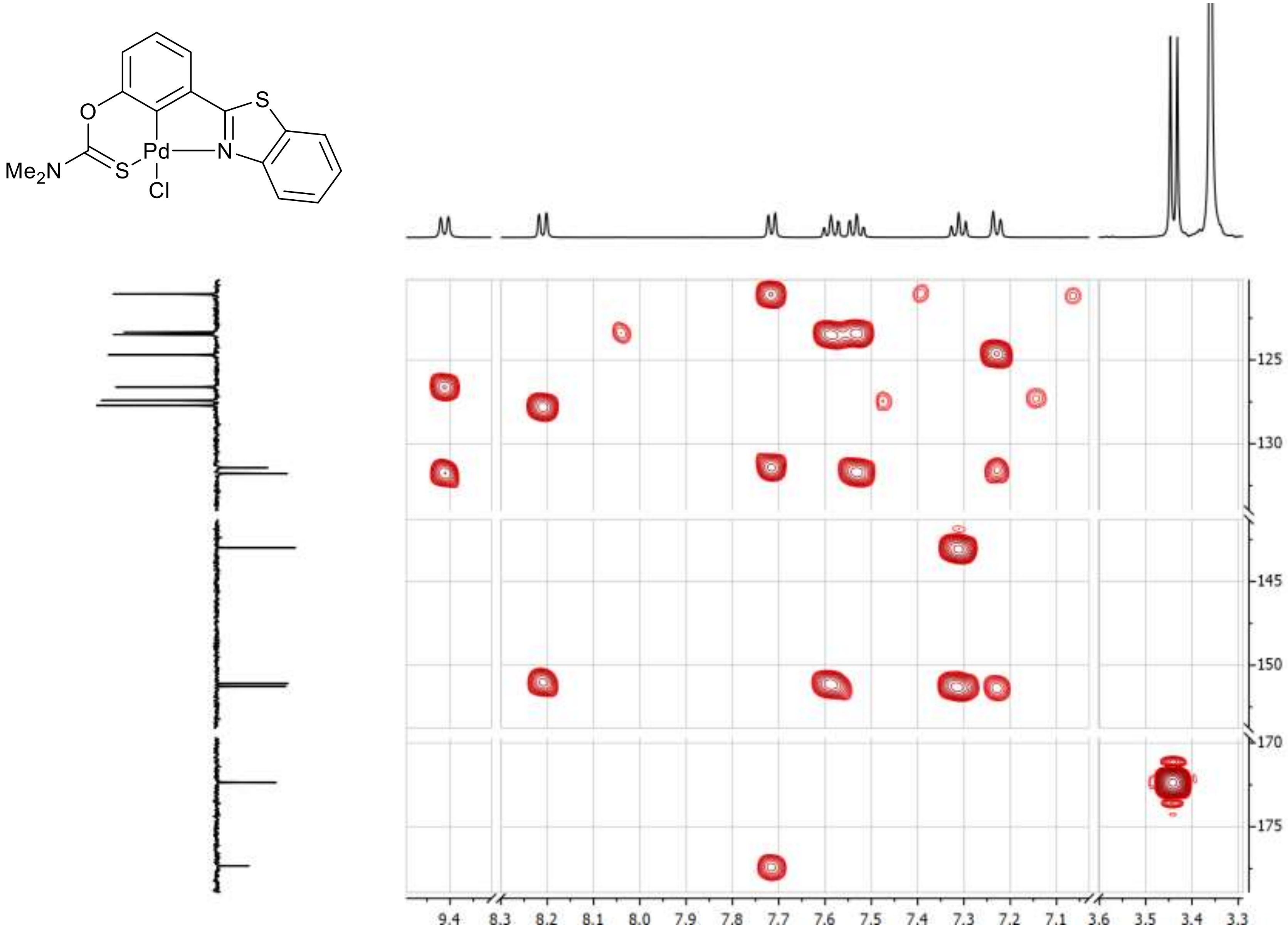


**Figure S18.** Extended fragments of the $^{1}H-^{13}C$ HMBC spectrum of complex **2a** ($(CD_3)_2SO$)

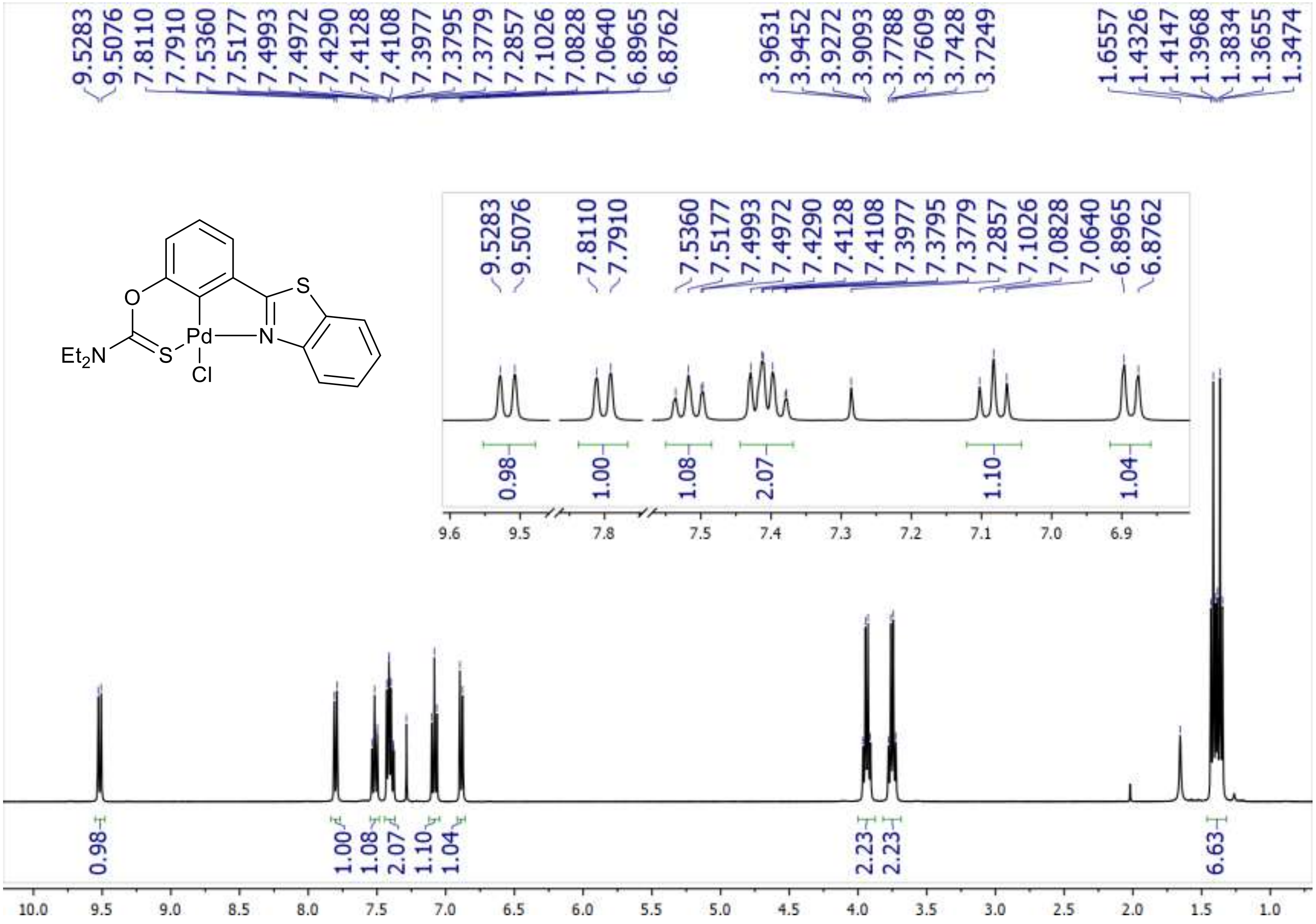

**Figure S19.** $^1$H NMR spectrum of complex **2b** (400.13 MHz, $CDCl_3$)

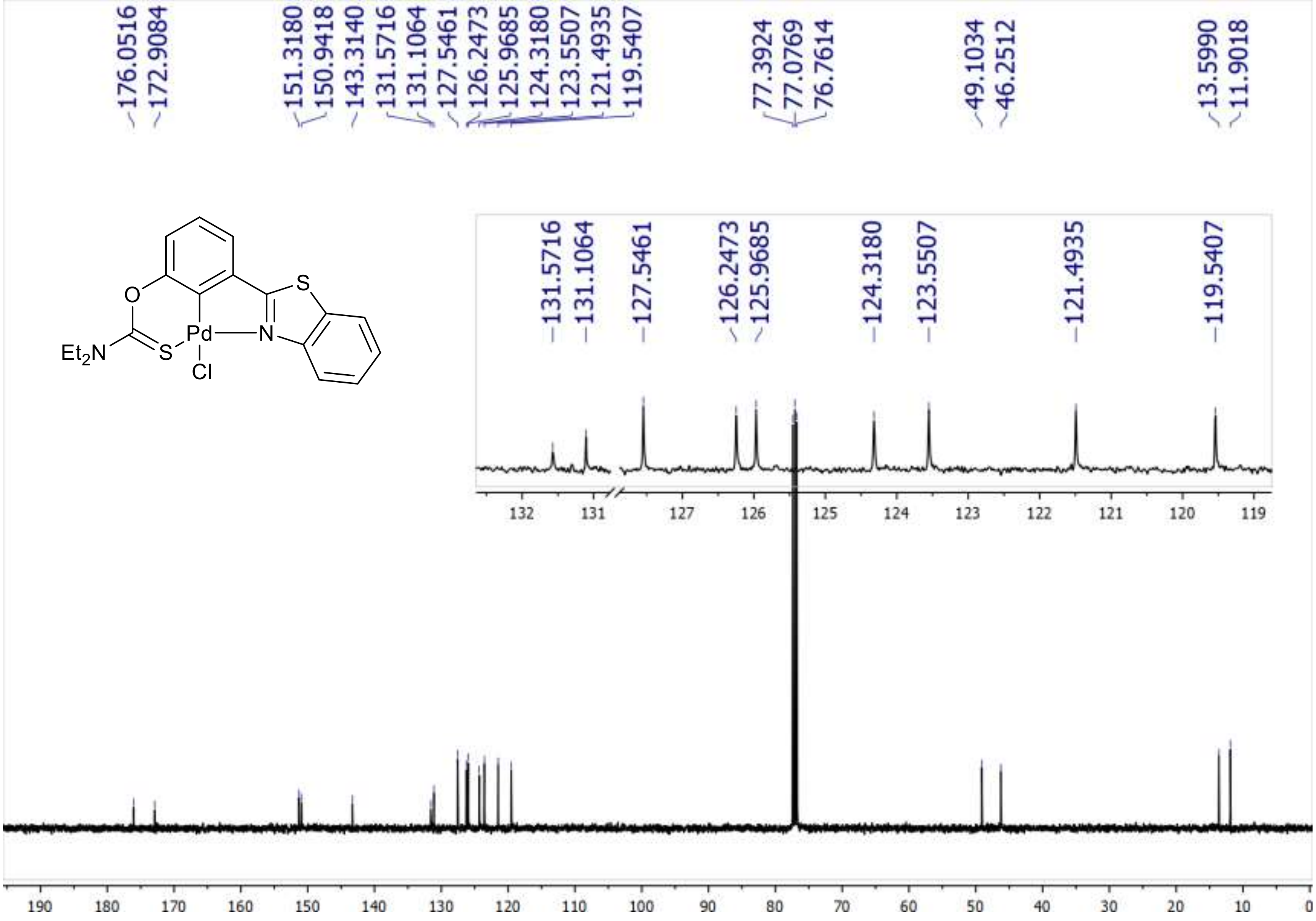


**Figure S20.** $^{13}C\{^1H\}$ NMR spectrum of complex **2b** (100.61 MHz, $CDCl_3$)

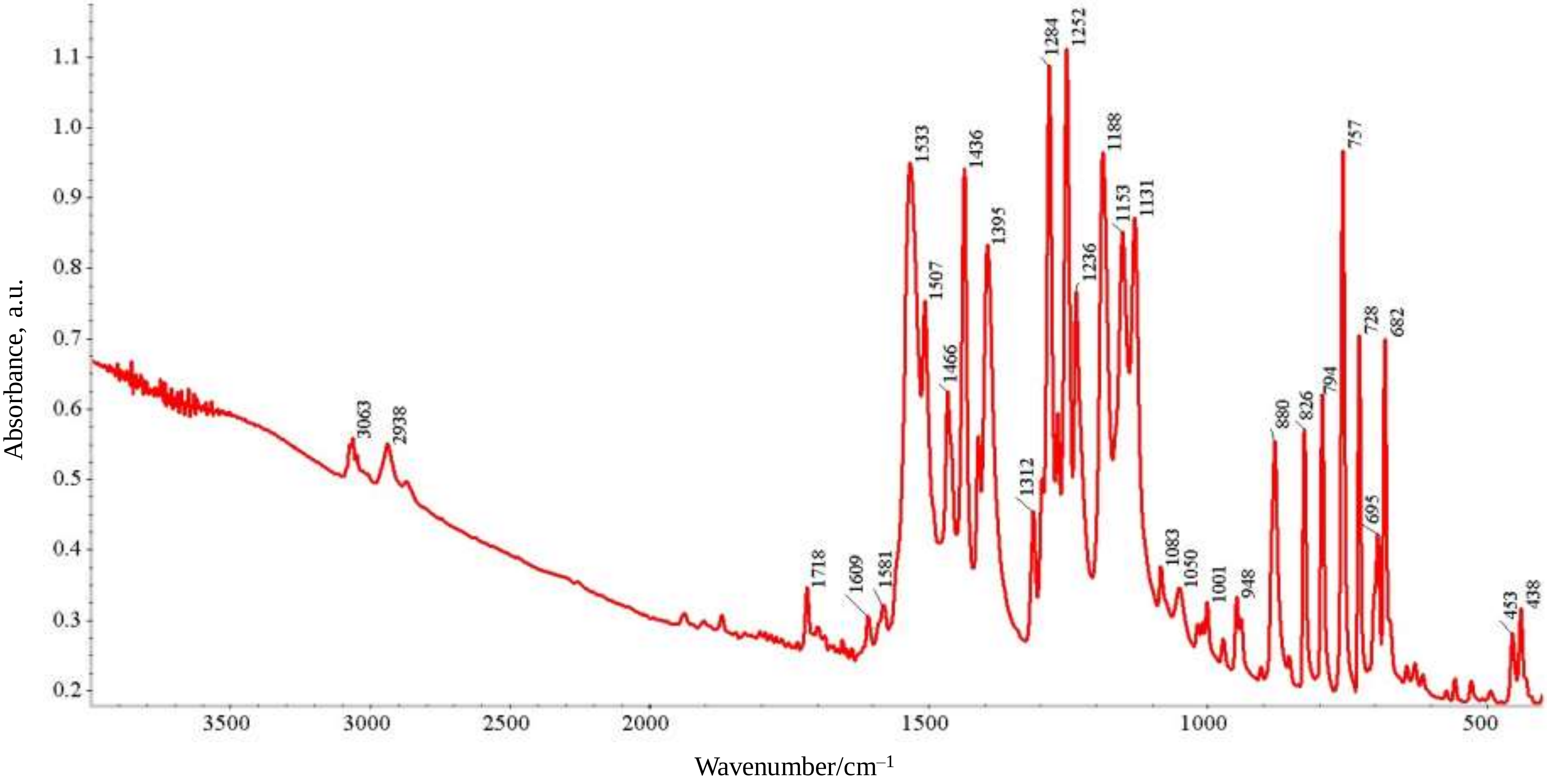


**Figure S21.** IR spectrum of ligand **1a**

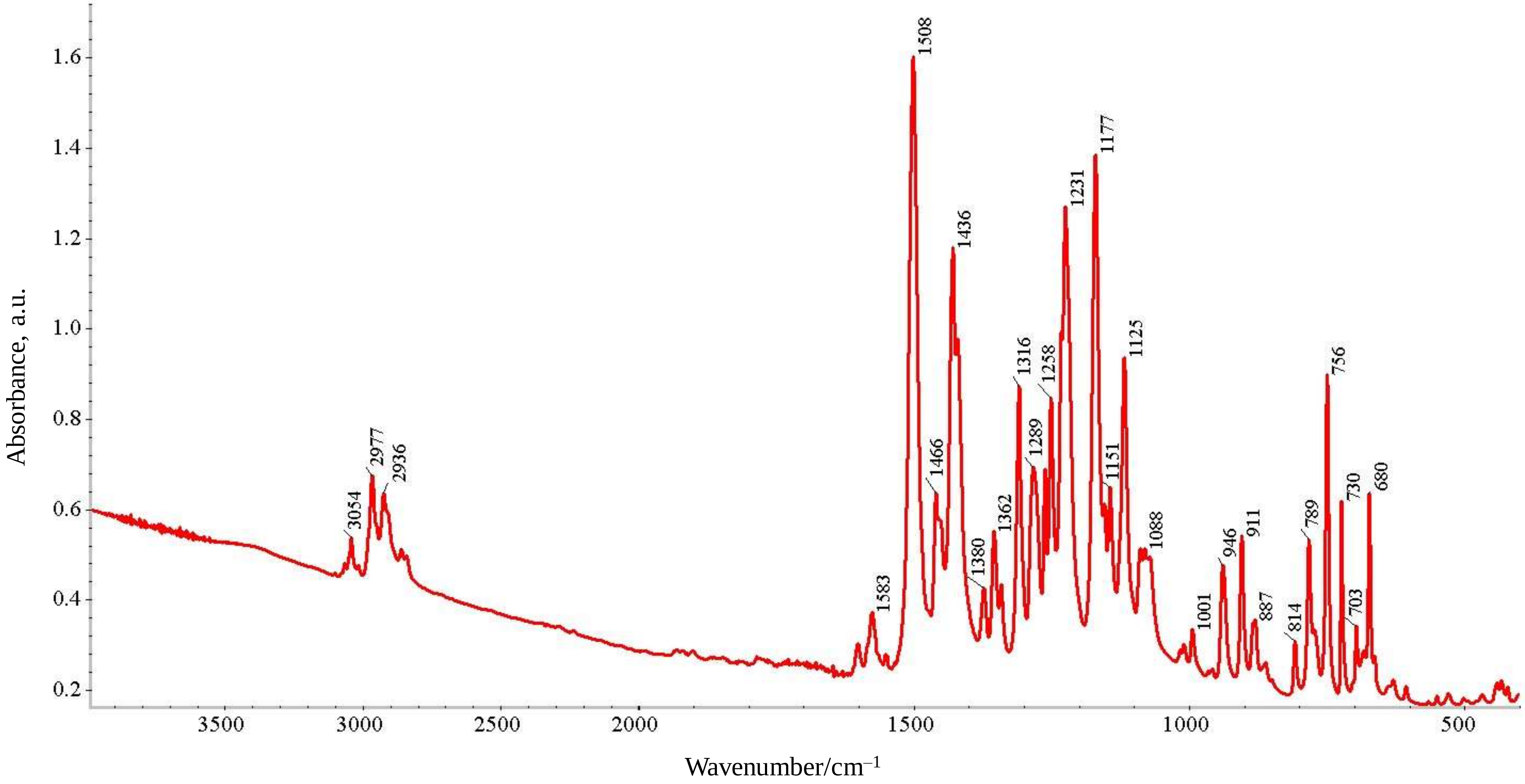


**Figure S22.** IR spectrum of ligand **1b**

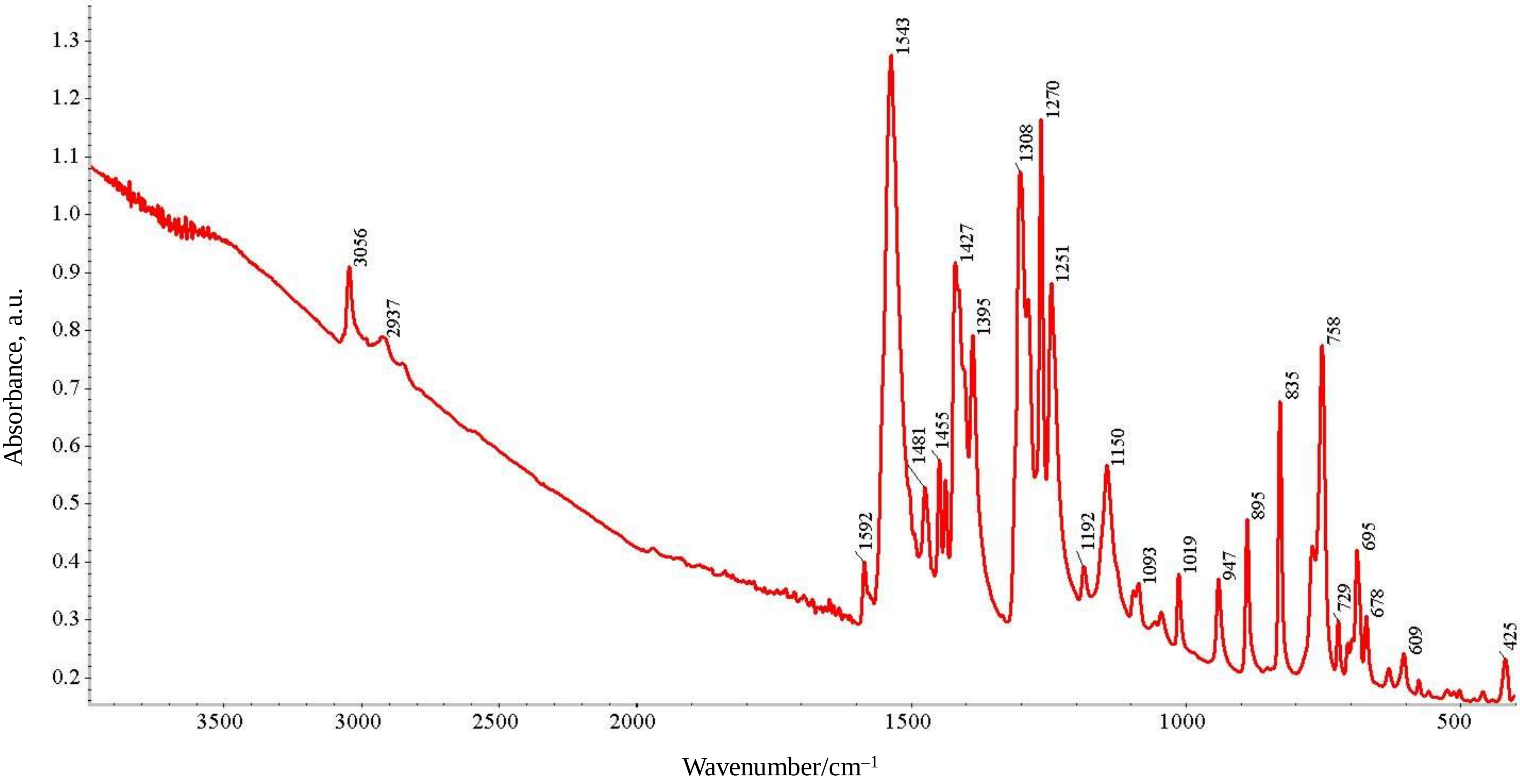


**Figure S23.** IR spectrum of complex **2a**

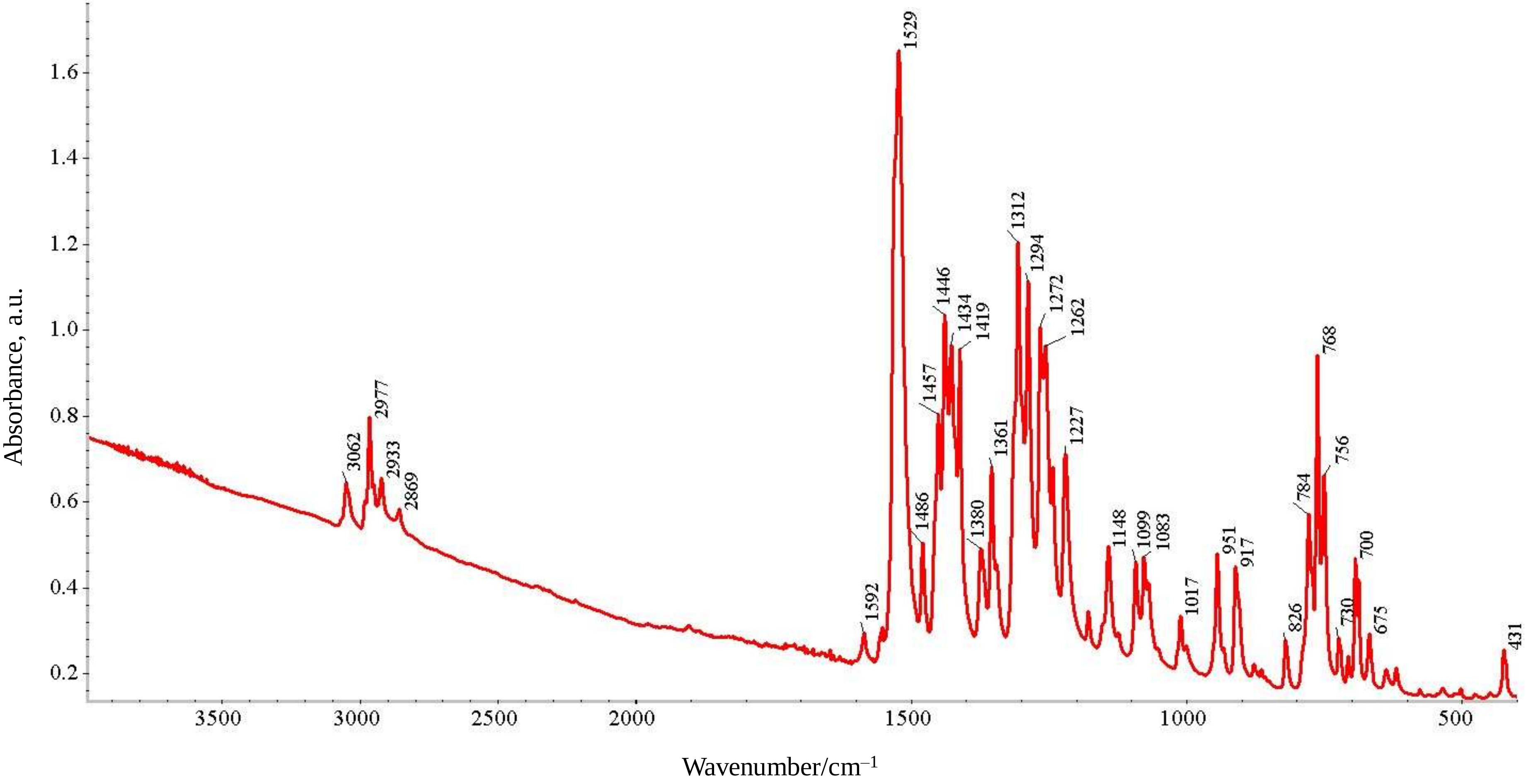


**Figure S24.** IR spectrum of complex **2b**

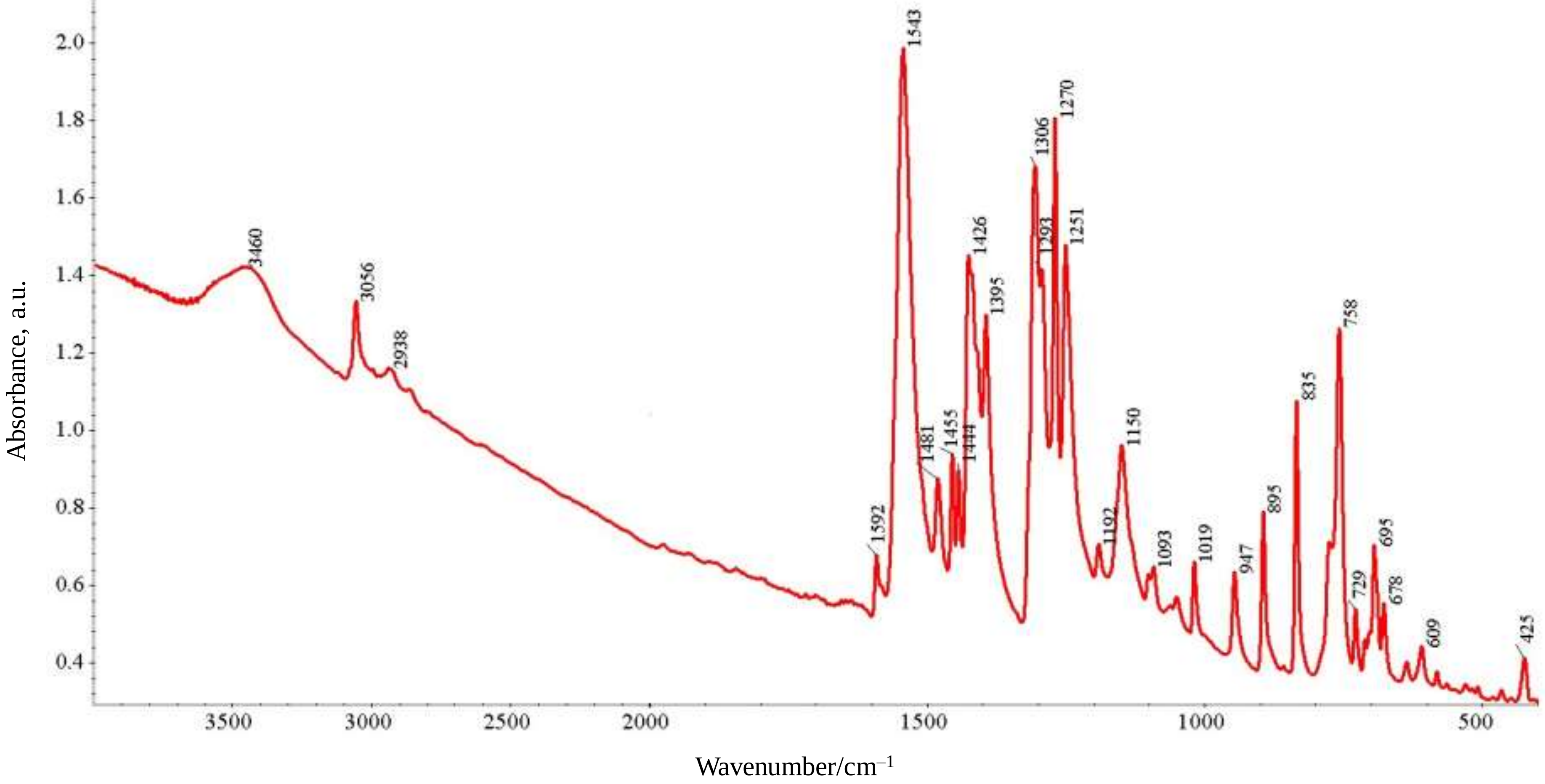


**Figure S25.** IR spectrum of a light yellow solid obtained after heating of the ground mixture of ligand **1a** and $PdCl_2(NCPh)_2$

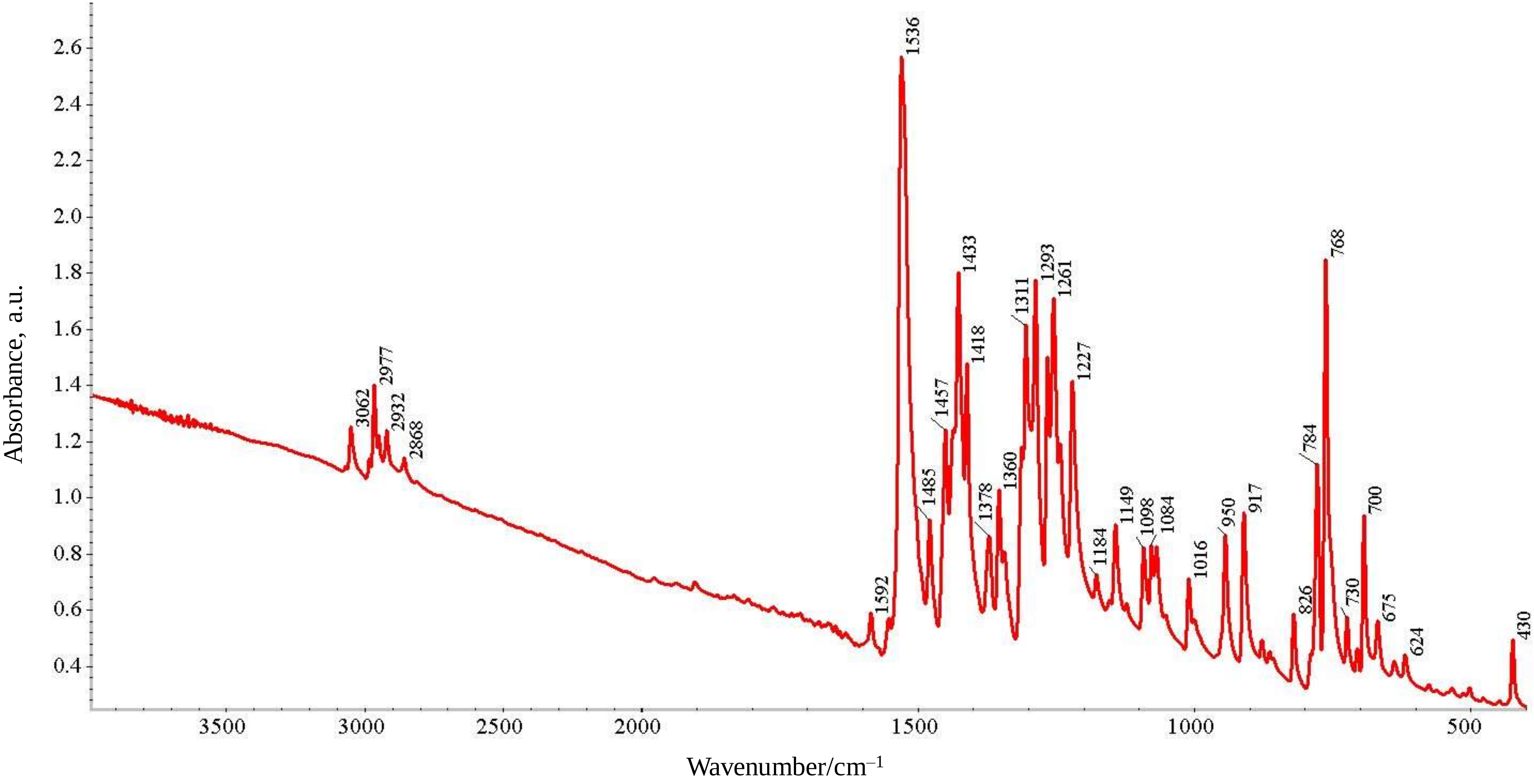


**Figure S26.** IR spectrum of a light yellow solid obtained after heating of the ground mixture of ligand **1b** and $PdCl_2(NCPh)_2$

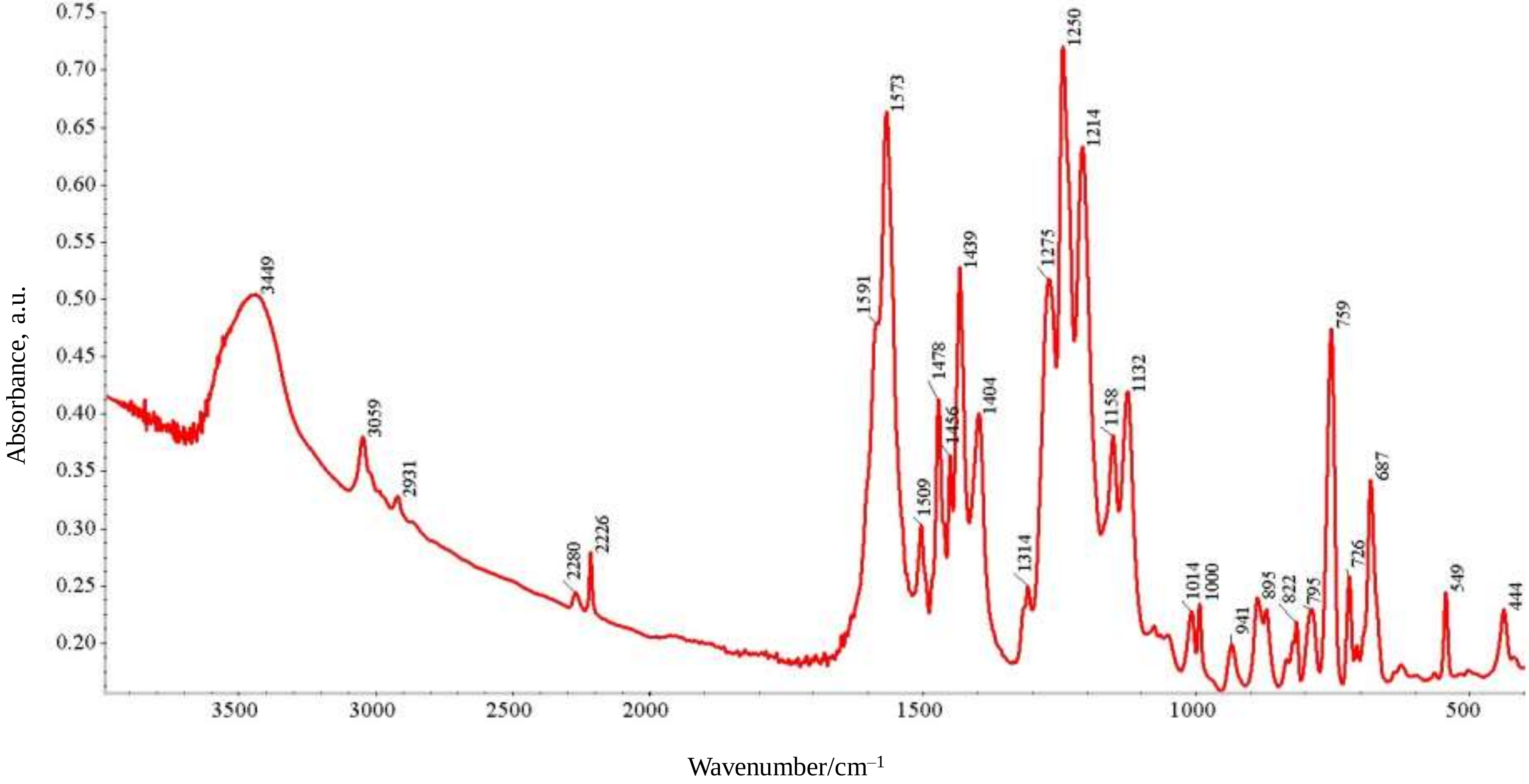


**Figure S27.** IR spectrum of an orange slightly oily powder obtained by grinding ligand **1a** with $PdCl_2(NCPh)_2$

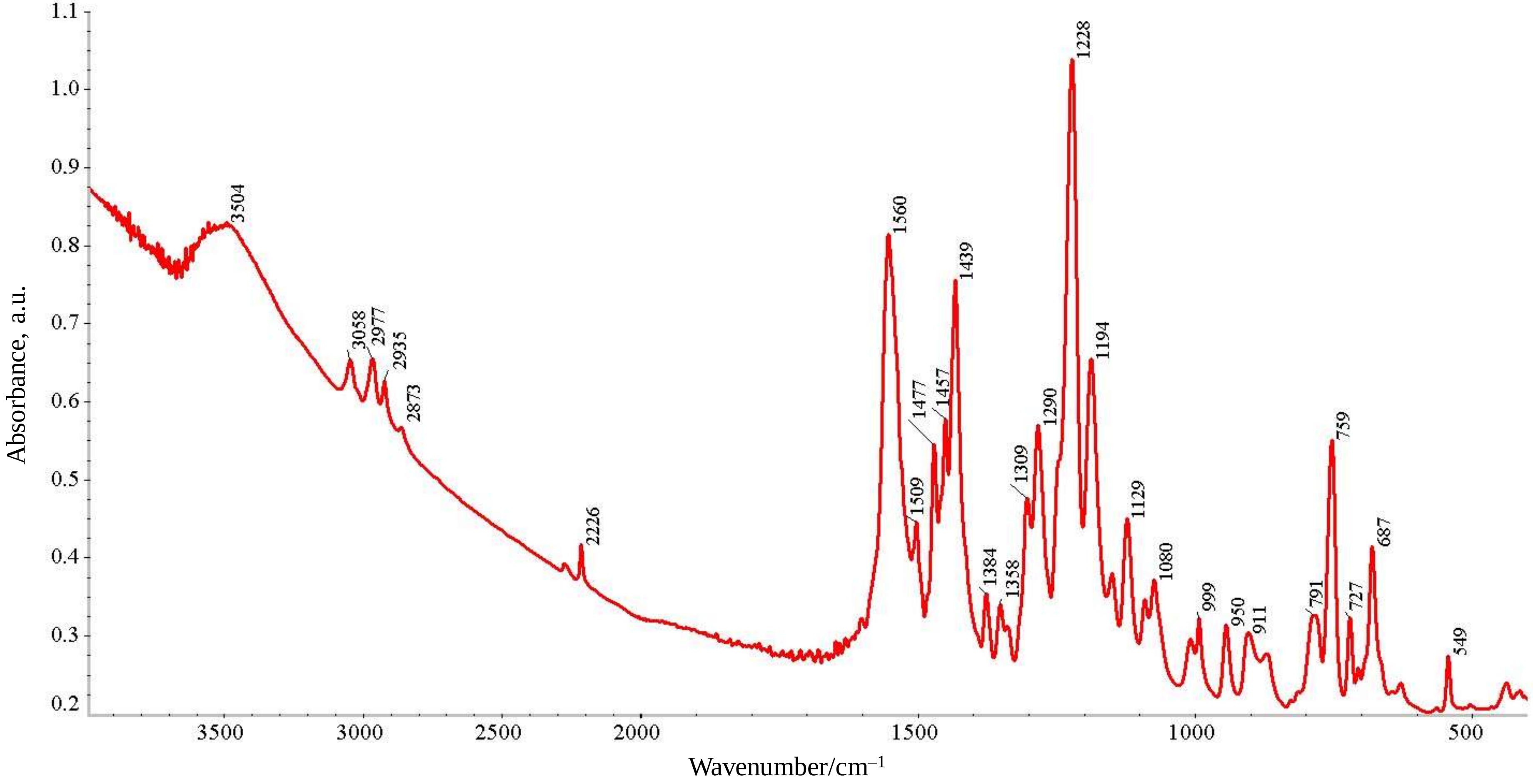


**Figure S28.** IR spectrum of a brown slightly oily powder obtained by grinding ligand **1b** with $PdCl_2(NCPh)_2$

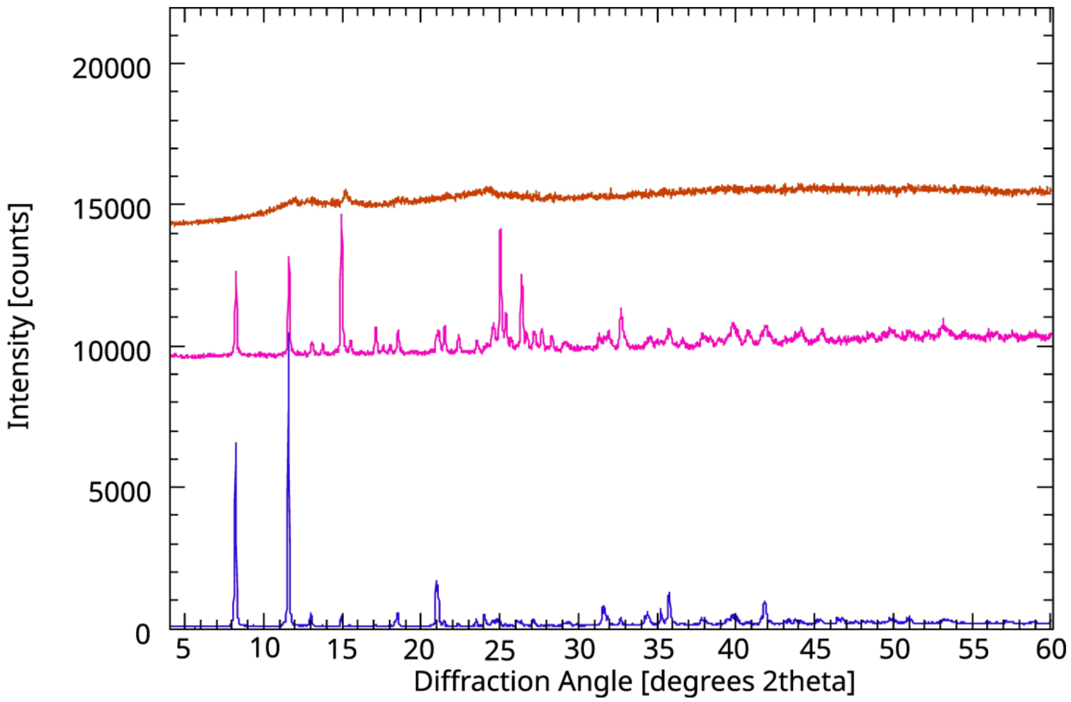


**Figure S29.** XRD patterns for an oily solid residue obtained by grinding ligand **1a** with $PdCl_2(NCPh)_2$ in a mortar (red) and a light yellow powder obtained after its heating (magenta), as well as an authentic sample of pincer complex **2a** derived from the conventional solution-based synthesis (blue)

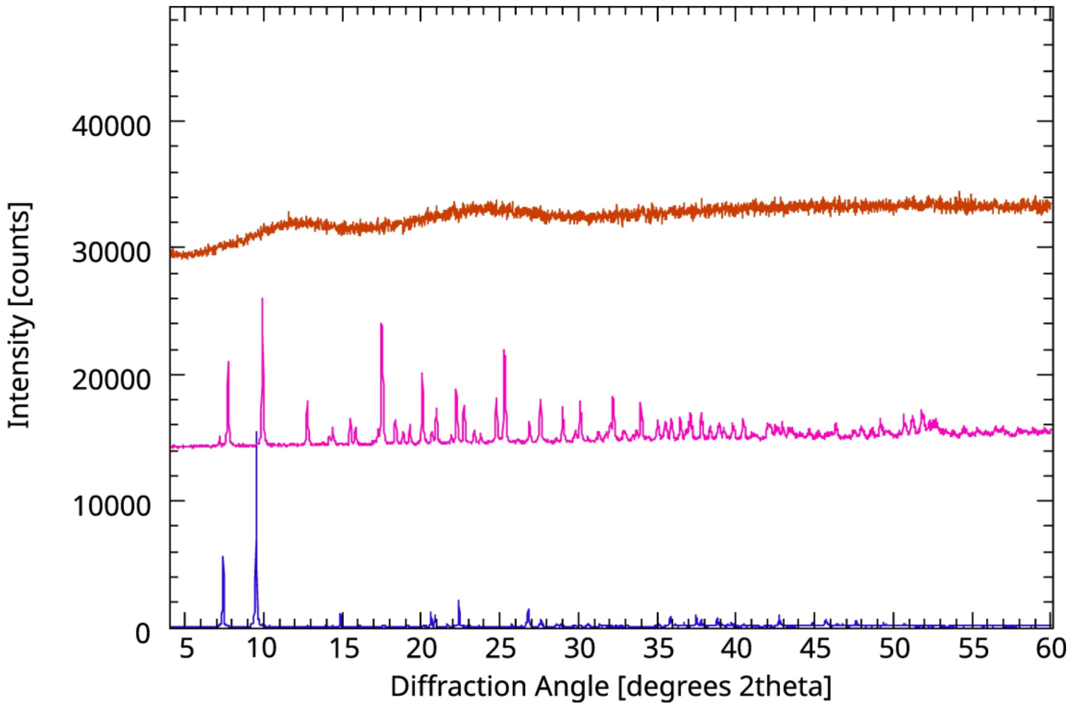


**Figure S30.** XRD patterns for an oily solid residue obtained by grinding ligand **1b** with $PdCl_2(NCPh)_2$ in a mortar (red) and a light yellow powder obtained after its heating (magenta), as well as an authentic sample of pincer complex **2b** derived from the conventional solution-based synthesis (blue)

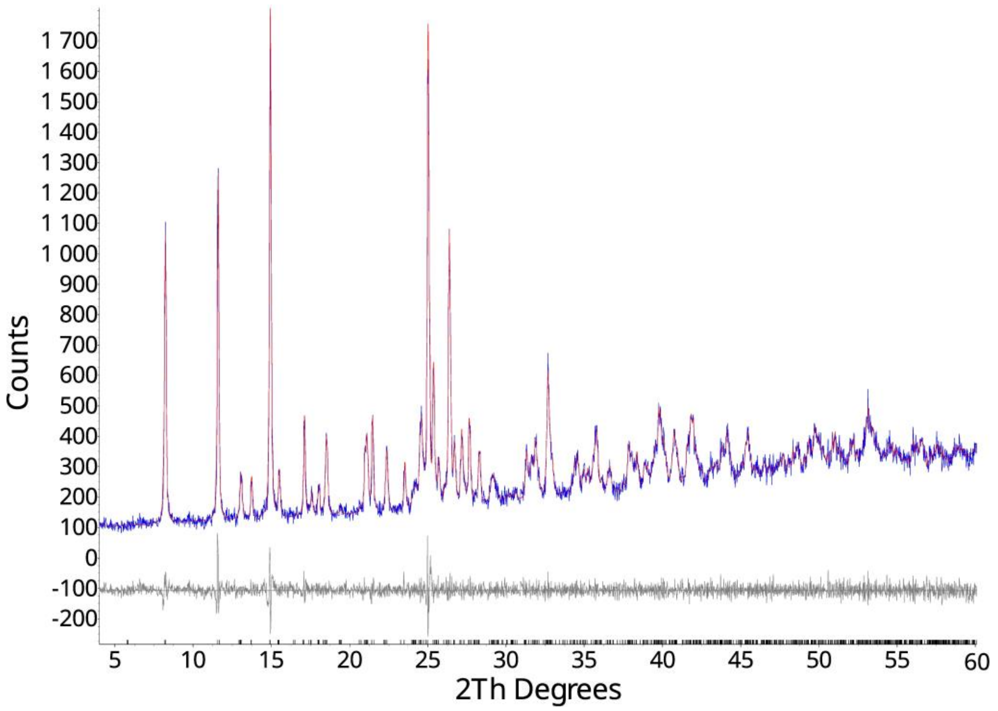


**Figure S31.** XRD pattern and the results of the Pawley fit for a solid sample obtained by heating of the ground mixture of ligand **1a** and $PdCl_2(NCPh)_2$. The experimental, calculated, and difference curves are shown by blue, red and grey colors, respectively

Cell parameters: a = 21.599(6), b = 7.1252(9), c = 21.464(5) Å, β = 89.451(12), V = 3303.1(13) $Å^3$, space group $P2_1/n$. Note that the value of a cell volume allows one to assume that, in this case, a unique part of the unit cell contains two molecules.

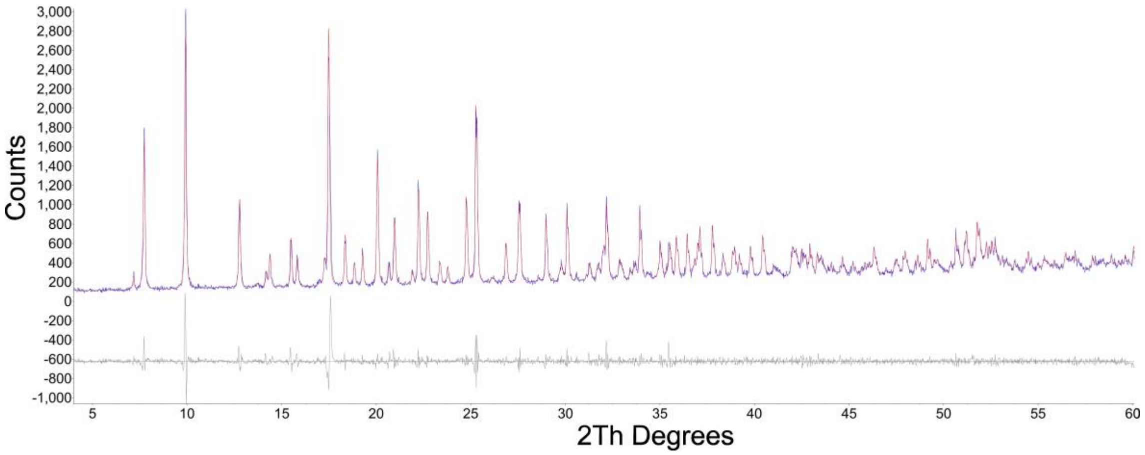


**Figure S32.** XRD pattern and the results of the Pawley fit for a solid sample obtained by heating of the ground mixture of ligand **1b** and $PdCl_2(NCPh)_2$. The experimental, calculated, and difference curves are shown by blue, red and grey colors, respectively

Cell parameters: a = 12.9056(10), b = 24.6515(14), c = 5.7489(7) Å, β =90.125(11)°, V = 1829.0(3) $Å^3$, space group $P2_1/c$.

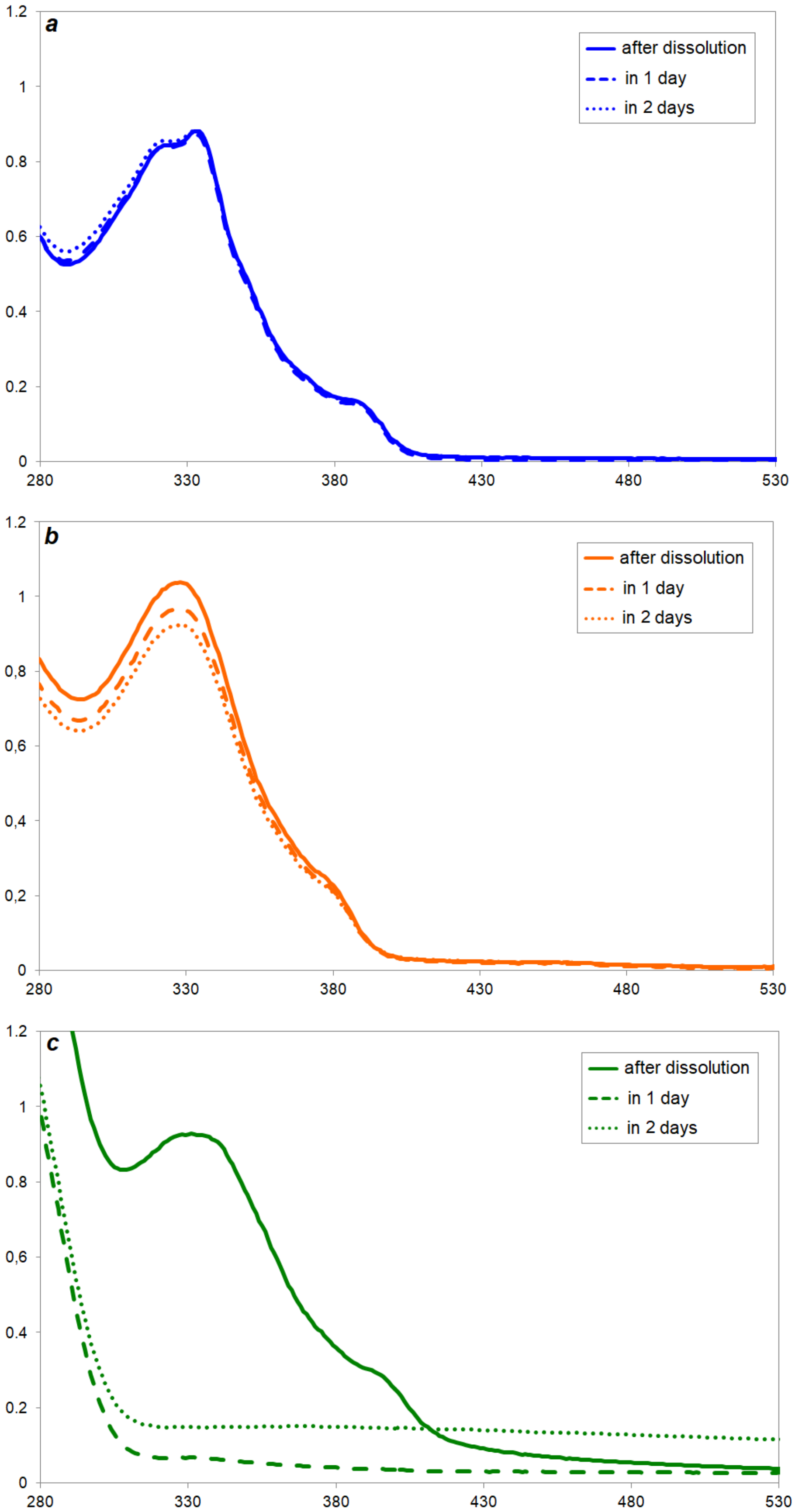


**Figure S33.** UV-Vis spectra of complex **2b** in DMSO (***a***), DMSO–deionized water (1:1) (***b***), and DMSO–PBS (1:1) (***c***) registered immediately after dissolution, in one or two days

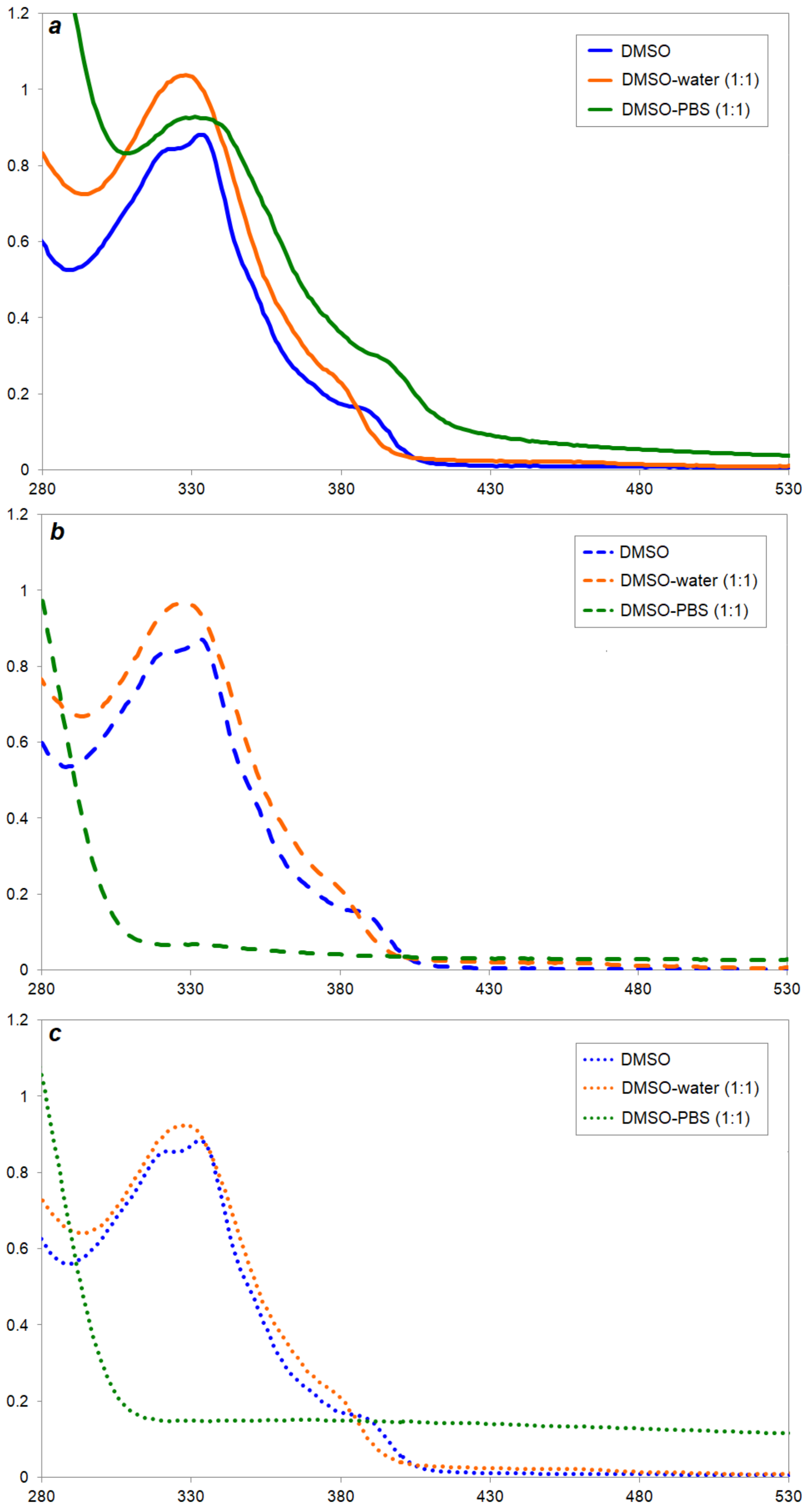


**Figure S34.** UV-Vis spectra of complex **2b** in DMSO, DMSO–deionized water (1:1), and
DMSO–PBS (1:1) registered immediately after dissolution (***a***), in one (***b***) or two (***c***) days

Note: a reduction in the absorbance in the case of the DMSO–PBS (1:1) solution in time is connected with the gradual precipitation of the complex under investigation. To confirm its identity, the resulting precipitate was collected by filtration, dried in air, dissolved in neat DMSO, and immediately analyzed by UV-Vis spectroscopy (see Fig. S35).

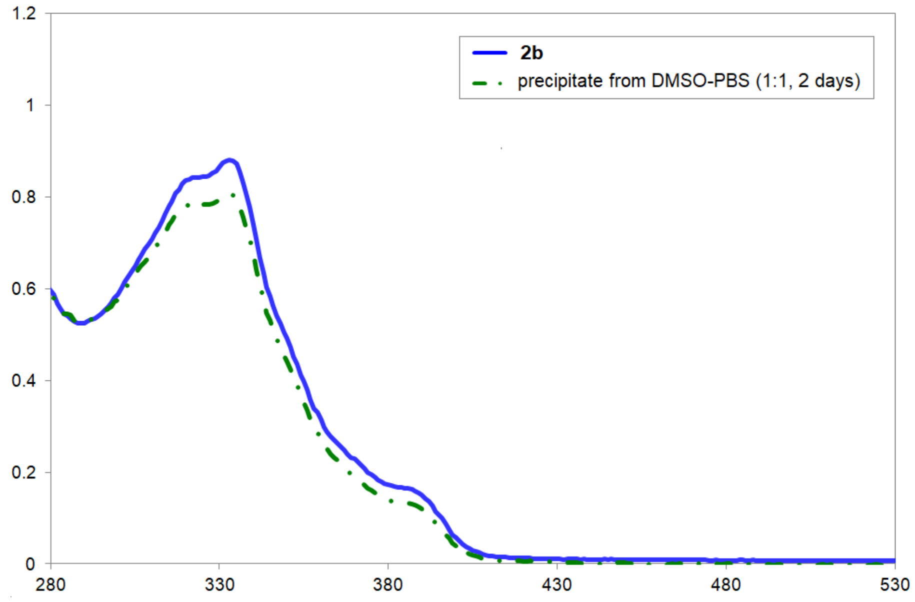


**Figure S35.** UV-Vis spectra of complex **2b** in DMSO and the precipitate from DMSO–PBS (1:1, 2 days) in DMSO registered immediately after dissolution

**Table S1.** Crystal data and structure refinement parameters for complex **2a**

| | |
|---|---|
| Empirical formula | $C_{16}H_{13}ClN_2OPdS_2$ |
| Formula weight | 553.98 |
| T, K | 200 |
| Crystal system | Triclinic |
| Space group | $P\bar{1}$ |
| Z | 6 |
| a, Å | 15.345(11) |
| b, Å | 16.270(12) |
| c, Å | 16.298(12) |
| α, ° | 78.393(9) |
| β, ° | 63.830(8) |
| γ, ° | 63.562(8) |
| V, Å$^3$ | 3270(4) |
| $D_{calc}$ (g cm$^{-1}$) | 1.688 |
| Linear absorption, μ (cm$^{-1}$) | 11.89 |
| F(000) | 1692 |
| 2θmax, ° | 50 |
| Reflections measured | 22703 |
| Independent reflections | 11317 |
| Observed reflections [I > 2σ(I)] | 4998 |
| Parameters | 709 |
| R1 | 0.1143 |
| wR2 | 0.3958 |
| GOF | 1.041 |
| $\Delta\rho_{max}/\Delta\rho_{min}$ (e Å$^{-3}$) | 1.972/–2.161 |
| CCDC | 2311540 |
